# Probing Folate-Responsive and Stage-Sensitive Metabolomics and Transcriptional Co-Expression Network Markers to Predict Prognosis of Non-Small Cell Lung Cancer Patients

**DOI:** 10.3390/nu15010003

**Published:** 2022-12-20

**Authors:** Yu-Shun Lin, Yen-Chu Chen, Tzu-En Chen, Mei-Ling Cheng, Ke-Shiuan Lynn, Pramod Shah, Jin-Shing Chen, Rwei-Fen S. Huang

**Affiliations:** 1 Department of Nutritional Science, Fu Jen Catholic University, New Taipei City 242062, Taiwan; 2 Metabolomics Core Laboratory, Healthy Aging Research Center, Chang Gung University, Taoyuan 33302, Taiwan; 3 Department of Mathematics, Fu Jen Catholic University, New Taipei City 242062, Taiwan; 4 Praexisio Taiwan Inc., New Taipei City 22180, Taiwan; 5 Division of Thoracic Surgery, Department of Surgery, National Taiwan University Hospital, Taipei 100225, Taiwan

**Keywords:** tumour folate, target metabolomics, transcriptional profile, WGCNA, non–small-cell lung cancers

## Abstract

Tumour metabolomics and transcriptomics co-expression network as related to biological folate alteration and cancer malignancy remains unexplored in human non-small cell lung cancers (NSCLC). To probe the diagnostic biomarkers, tumour and pair lung tissue samples (n = 56) from 97 NSCLC patients were profiled for ultra-performance liquid chromatography tandem mass spectrometry (UPLC/MS/MS)-analysed metabolomics, targeted transcriptionomics, and clinical folate traits. Weighted Gene Co-expression Network Analysis (WGCNA) was performed. Tumour lactate was identified as the top VIP marker to predict advance NSCLC (AUC = 0.765, Sig = 0.017, CI 0.58–0.95). Low folate (LF)-tumours vs. adjacent lungs displayed higher glycolytic index of lactate and glutamine-associated amino acids in enriched biological pathways of amino sugar and glutathione metabolism specific to advance NSCLCs. WGCNA classified the green module for hub serine-navigated glutamine metabolites inversely associated with tumour and RBC folate, which module metabolites co-expressed with a predominant up-regulation of LF-responsive metabolic genes in glucose transport (*GLUT1*), de no serine synthesis (*PHGDH*, *PSPH*, and *PSAT1*), folate cycle (*SHMT1/2* and *PCFR*), and down-regulation in glutaminolysis (*SLC1A5*, *SLC7A5*, *GLS*, and *GLUD1*). The LF-responsive WGCNA markers predicted poor survival rates in lung cancer patients, which could aid in optimizing folate intervention for better prognosis of NSCLCs susceptible to folate malnutrition.

## 1. Introduction

Folate is an essential nutrient with a diverse metabolic role of supporting normal cellular function and health [1]. Folate-mediated one-carbon metabolism contributes to multiple biochemical pathways of nucleotide biosynthesis, amino acid homeostasis [2], redox homeostasis [3,4], and epigenetic regulation to support normal growth and mass expansion of organism [5]. Cellular and animal studies has shown that folate restriction enhanced migration, invasiveness, and anchorage-independent oncospheroid formation in human colon [6,7], lungs [8], and human breast cancers [9]. However, supplemental high folate did not offset folate deprived-metabolic perturbation, but further promoted metastatic potentials of lung cancer cells and mouse tumours [10,11]. Human studies have examined association of dietary and blood folate status with lung cancer, but results were inconclusive [12,13,14,15]. Regardless of the mixed outcome of preclinical and clinical studies on suboptimal folate-associated cancers, the direct link of altered tumour folate availability to cancer malignancy biomarkers remains rarely explored in human.

Recent advances propose the reprogramming of cancer metabolism as the hallmark of cancer malignancies [16], in particular for lung cancer (LC), as the leading cause of cancer-related deaths worldwide with high prevalence and low survival rates [17]. Studies on cellular and rodent models had documented lactate metabolic phenotype of LC [18] associated with malignancy transformation [19], known for aerobic glycolysis as “Warburg effect” [20]. Multiple altered metabolic pathways in tumours are made even more complex by the diverse genome-wide backgrounds, various metabolite requirements of tumours, and differential pathological course at malignancy progress [21]. Crosstalk network between transcriptional regulators and metabolism results in metabolically heterogeneous entity of cancers, which is remodelled by metabolite nutrient availability and mobilization in the tumour microenvironment [22,23,24]. Thus far, it remains unexplored whether limited folate metabolite availability, the most prevalent folate malnutrition prone to cancer cachexia and chemotherapy [25], may remodel tumours’ genetic and metabolic crosstalk network to impact cancer malignancy transformation. This knowledge gap highlights the urgent need to understand folate metabolism in spontaneously arising human tumours. Studies to probe folate-responsive and advance stage-sensitive tumour markers are warranted for optimizing folate intervention and better prognosis of non-small cell lung cancer (NSCLC), accounting for 80% of LC types [26].

Accordingly, the aims of the study were to explore tumour metabolomics and transcriptomics co-expression network markers as to biological folate alteration and cancer stage in NSCLC. Tumour and pair lung tissue samples (n = 56) from NSCLC patients were collected and analysed for metabolomics markers by ultra-performance liquid chromatography tandem mass spectrometry (UPLC/MS/MS), and for transcriptomics profiling by RT-PCR. Comprehensive folate-responsive and stage-sensitive metabolic and genetic network analysis was performed by Weight Gene Co-expression Network Analysis (WGCNA). Multiple linear regression and the Kaplan–Meier survival curves were conducted to identify folate-sensitive tumour markers to predict overall survival of LC patients. The results are discussed.

## 2. Materials and Methods

### 2.1. Patient Cohort and Paired Tumours Tissue Acquisition

The study cohort consisted of consecutive patients with lung cancer diagnosed at Clinical Thoracic Surgery Department in National Taiwan University Hospital (NTUH), Taipei, Taiwan, between 2017 and 2020. The criteria for inclusion were (1) low-dose computed tomography and histopathologic diagnosis of NSCLC lesions; (2) None received either chemotherapy or radiotherapy before surgery; (3) no serious complication of liver diseases, cardiovascular diseases, kidney diseases, diabetes, and other cancers. Ninety-seven NSCLC patients were included into the cohort study according to the designated criteria. The Joint Ethical Committee of NTUH and Fu Jen Catholic University Hospital approved the study. Written informed consent was secured from all the study subjects. 

The institutional review board (IRB) protocol was approved by the ethics committee at Fu Jen Catholic University Hospital (ethic approval code: C105018), and National Taiwan University Hospital (ethic approval code: 201701123RINC). Thirty patients approved the IRB protocol with their denoting consent for paired tumours tissue acquisition. Residual tumours and adjacent lung tissues were resected by surgeons, and were aliquoted into 1.5 mL Nunc vials, immediately placed in liquid nitrogen, barcoded, and stored at −80 °C for subsequent analysed. After surgery, cancer malignancy was diagnosed by pathologists according to the International Association for the Study of Lung Cancer, American Thoracic Society, and European Respiratory Society (IASLC/ATS/ERS) classification [27].

### 2.2. Basic Data and Blood Sample Collection

Basic anthropometric and dietary intake data of 97 NSCLCs patients were collected by dietitians. The dietary folate intake was calculated by a specialized quantitative food frequency questionnaire specifically designed for the assessment of folate with reference to the previously described semiquantitative FFQ for folate [28]. Body mass index (BMI) was calculated by body weight in kilograms divided by the square of height in meters. Prior to the surgery, fasting blood samples were collected, chilled, and transported to the Biomedicine Laboratory at Fu Jen Catholic University. Plasma and red blood cells (RBC) samples were immediately separated upon arrival and were stored at −80 °C until further analysis.

### 2.3. Determination of Clinical Folate Markers 

Plasma folate and homocysteine levels were measured using commercially available kits by fluorescence polarization immunoassay (Becton Dickinson, Franklin Lakes, NJ, USA) on an Abbott 130 AxSYM system (Becton Dickinson). Tissue folate was quantified using a microbiological assay by glycerol-protected Lactobacillus casei (BCRC^®^10697) in 96-well microtiter plates as previously described elsewhere [29]. Lymphocytic DNA was extracted using standard proteinase K digestion and the phenol–chloroform extraction procedure. The MTHFR C677T polymorphism was determined through RT-PCR and melting curve analysis by using a LightCycler instrument (Light-Cycler, Roche Diagnostics, Mannheim, Germany) as previously described [30]. Bisulfite modification of lymphocytic DNA was performed using the EpiTect PCR Control DNA set (Qiagen, Hilden, Germany), and the high-resolution melt-based PCR method was used to measure DNA methylation. Primers used for LINE-1 were F 5′-GCG AGG TAT TGT TTT ATT TGG GA-3′ and R 5′-CGC CGT TTC TTA AAC C-3′ to encompass eight CpG islands between primers and yield 141 bp of amplicon size. RT-PCR was conducted by use of a LightCycler instrument (Light-Cycler, Roche Diagnostics, Mannheim, Germany).

### 2.4. Transcripomics Analysis

Total RNA was extracted with an RNA REzol C&T reagents kit (Protech Technology, Taipei, Taiwan), and RT-PCR was conducted as previously described [6]. Briefly, 1 µg of each sample was reverse-transcribed in an MMLV Reverse Transcriptase 1st-Strand cDNA Synthesis Kit. Gene transcripts were amplified with specific primers. The cycling conditions included an initial phase of 2 min at 50 °C; 10 min at 95 °C; then 40 cycles of 10 s at 95 °C, 0.5 min at 60 °C, and 10 s at 72 °C. Amplified complementary DNA was quantified using the StepOnePlus Real-Time PCR system (Applied Biosystems, Waltham, MA, USA). 

### 2.5. UPLC/MS/MS Quantitative Metabolomics Analysis

Targeted UPLC/MS/MS metabolites analysis was used to detect the metabolites concentration in lung and tumour tissue. Frozen tumour and matched adjacent nontumor lung tissues (100 mg) were placed in a homogenization tube containing ceramic beads with a diameter of 1.4 mm (Precellys, Bertin Technologies, France). Ice-cold 50% methanol was added to each tube for tissue homogenization by Percellys 24 homogenizer (PEQLAB Biotechnology GmbH, Germany). The homogenized samples were then centrifuged at 4 °C with 12,000× *g* for 30 min, and acetonitrile was added to the supernatant for protein precipitation. After centrifugation, the supernatant was dried under nitrogen gas, dissolved in water, and centrifuged to remove debris. The supernatant was analysed using Waters ultra-high-performance liquid chromatography coupled with Waters Xevo TQ XS Mass Spectrometer (Waters Corp., Milford, MA, USA). Mass spectrometer was operated in negative with multiple reaction monitoring mode. Major fragment patterns of each analyte were determined with tuning. The chromatographic separation was achieved on a BEH C18 (100 × 2.1 mm, particle size of 1.7 um; Waters Corp.) at 45 °C with elute A (water with 10 mM tributylamine and 15 mM acetic acid) and eluent B (50% acetonitrile with 10 mM tributylamine and 15 mM acetic acid), and the flow rate was set at 0.3 mL/min. The gradient profile was as follows: isogradient 4% B, 6 min; linear gradient 4–50% B, 0.1 min; 50–60% B, 2.9 min; 60–100% B, 0.8 min; and keep 2.2 min. The column was then re-equilibrated for 3 min. Chromatographic separation was performed on a Waters ACQUITY BEH C18 column (2.1 mm × 100 mm × 1.7 µm, Waters corp.). QC samples (laboratory quality control cells) were prepared for analysed during the analytical runs after every 10th sample [31].

### 2.6. Enrichment Analysis

To identify significantly enriched pathway, metabolites in this study were performed using MetaboAnalyst 5.0 (http://www.metaboanalyst.ca, accessed on 21 December 2021) for enrichment analysis and visualization of the affected pathway. Metabolites set enrichment analysis was analysed based on the folate-responsive and stage-sensitive. Metabolites involved in the significantly enrichment pathways were identified based on Kyoto Encyclopaedia of Genes and Genomes database.

### 2.7. Metabolite and Genetic Correlation Network Analysis

A correlation network was used to visualize the relationship between metabolites and metabolites, and genes and metabolites. Debiased Sparse Partial Correlation (DSPC) algorithm was used to calculate partial correlation between metabolomics markers in folate-responsive, stage-sensitive, and LINE-1 levels. The correlation networks were mapped based on DSPC results using MetScape version 3.1.3 [32]. In genes and metabolites correlation network analysis, Pearson correlation was used to explore relationship between transcripts levels and metabolites signal intensity in tumour. The heatmap was constructed according to correlation results and performed using GraphPad Prism 9.

### 2.8. Survival Analysis of Public Data from Cancer Genomics Studies 

Data from The Cancer Genome Atlas Research Network (TCGA; Lung AC and Lung Squamous Cell Carcinoma Provisional sequenced tumours sample sets) were used to analyse ADSL and ATIC genetic alterations including mRNA expression z-scores (Microarray, threshold 2.0) by use of CBIPORTAL software (http://www.cbioportal.org/, accessed on 29 June 2021). Exploration from Human genetic Atlas datasets and two public metabolomics databases from the study of Luo et al. [33] and from the study of Qi et al. [34] were conducted. The effect of ADSL and ATIC genetic expression on LC patient prognoses was evaluated by Kaplan–Meier survival curves of NSCLC patients with low or high mRNA expression (KMPLOTTER; http://www.kmplot.com/analysis, accessed on 19 June 2021). A log-rank test was calculated to determine differences in overall survival using SPSS 11.5.0 for Windows (IBM Corp., Armonk, NY, USA). For selecting the oncotargets with a significant survival rate, *p* ≤ 0.05 was set as a cut off parameter.

### 2.9. General Statistical Analysis

Statistical analyses on clinical and biochemical data were performed using the statistical analysis system (SAS/STAT version 9.4, SAS Institute, Cary, NC, USA; SPSS Statistics, version 14, SPSS Inc., Chicago, IL, USA). Demographic and laboratory data of continuous variables were compared using one-way ANOVA (analysis of covariance) followed by a Duncan test. A chi-squared test was for categorial variables. Differences were considered to be statistically significant at *p* < 0.05. Multivariate linear regression models were constructed to evaluate the folate determinants of altered metabolomics signatures in tumours. The interaction of tumour folate and stage was tested using two-way ANOVA analysis. Survival analyses were performed on categorical variables of dichotomized metabolite abundances in SAS Enterprise Guide, version 4.2 (SAS Institute Inc., Cary, NC, USA), and all reported *p* values are two sided. Moreover, to assess the predictive power of biomarkers, receiver operating characteristic (ROC) analyses were performed through GraphPad Prism 9 (GraphPad Software, San Diego, CA, USA).

### 2.10. Metabolomics Statistical Analysis

Statistical mixed effects models, orthogonal partial least squares discriminant analysis (OPLS-DA) and network integration, were used to identify key cancer-associated metabolic perturbations in adenocarcinoma compared to non-malignant paired lung tissue. The metabolomics data files were subjected to extensive statistical analysis using MetaboAnalyst software (version 5.0; https://www.metaboanalyst.ca/, accessed on 21 December 2021) in order to identify the comparative and statistically distinguished metabolites for the search of FD-reprogramed NSCLC biomarkers. The data were normalized to unit scale to eliminate baseline differences in metabolism between tumours/adjacent lung tissues. The data on differential metabolites in paired tumours tissues or in blood metabolic conditions were validated at the univariate level using Student’s *t*-test (*p* < 0.05). A partial least square discriminant analysis (PLSDA) model was generated using statistically significant metabolites which, on external validation, provided high sensitivity (100%) and specificity (78.6%). Multivariate statistical analysis includes principal component analysis (PCA), the supervised OPLS-DA, the volcano plot analysis on fold change of metabolites, and variable importance in projection (VIP) as a measure of their relative influence on the model with a threshold > 2 through OPLS-DA. Partial Correlation heatmap was constructed by Spearman correlation coefficient analysis. Differences were considered to be statistically significant set at a level of probability of *p* < 0.05 and fold change > 2.

### 2.11. Weight Gene Co-Expression Network Analysis

WGCNA was performed to analysis metabolomic dataset according to Pei et al. [35] reported and constructed using R package “WGCNA”. Thirty-one metabolites were performed to explore the interactions between metabolites, and between metabolites and clinical traits (folate status, LINE-1, MTHFR, TMN stage, and gene expression) by WGCNA. First, we performed sample clustering to check for outliers. Second, correlation analysis was used to calculate the correlations between metabolites. Then, we used network topology analysis to determine the optimal soft threshold that can enhance the strong correlations between metabolites and punish the weak correlations between metabolites. The expression matrix was converted to obtain a topological overlap matrix. The soft threshold was chosen to be four, which complied with the scale-free network rules (Figure S3). After the minimum module size was set to three, hierarchical clustering was performed to generate co-expression modules [36]. At the same time, module eigengenes (MEs) in each module were also calculated. Finally, we evaluated the associations between ME and clinical traits to determine NSCLC-related modules for subsequent joint pathway analysis [37]. Hub-metabolites were constructed by CytoHubba in Cytoscape plug-in and verified by Maximal Clique Centrality method [38].

## 3. Results

### 3.1. Discover Stage-Sensitive Tumour Metabolomics Markers in Paired NSCLCs 

Basic and pathologic data of the paired NSCLCs are shown in Supplementary Table S1. Neither demographic (age, sex, BMI, and smoking) nor biological folate trait (dietary, plasma, RBC, lungs, and tumours) significantly differs between early (IA) and advance stage (IB-IVB) NSCLCs. 

**Figure 1:**
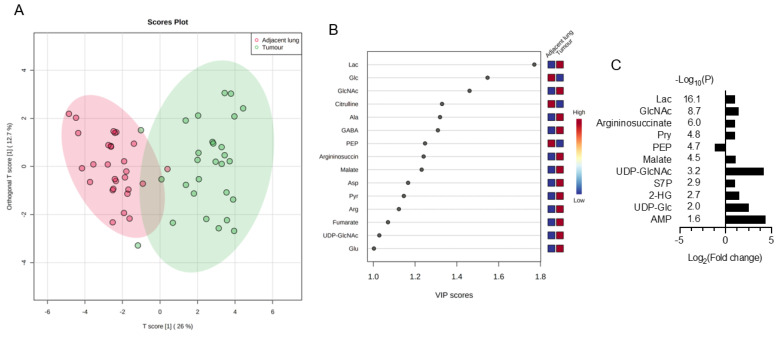

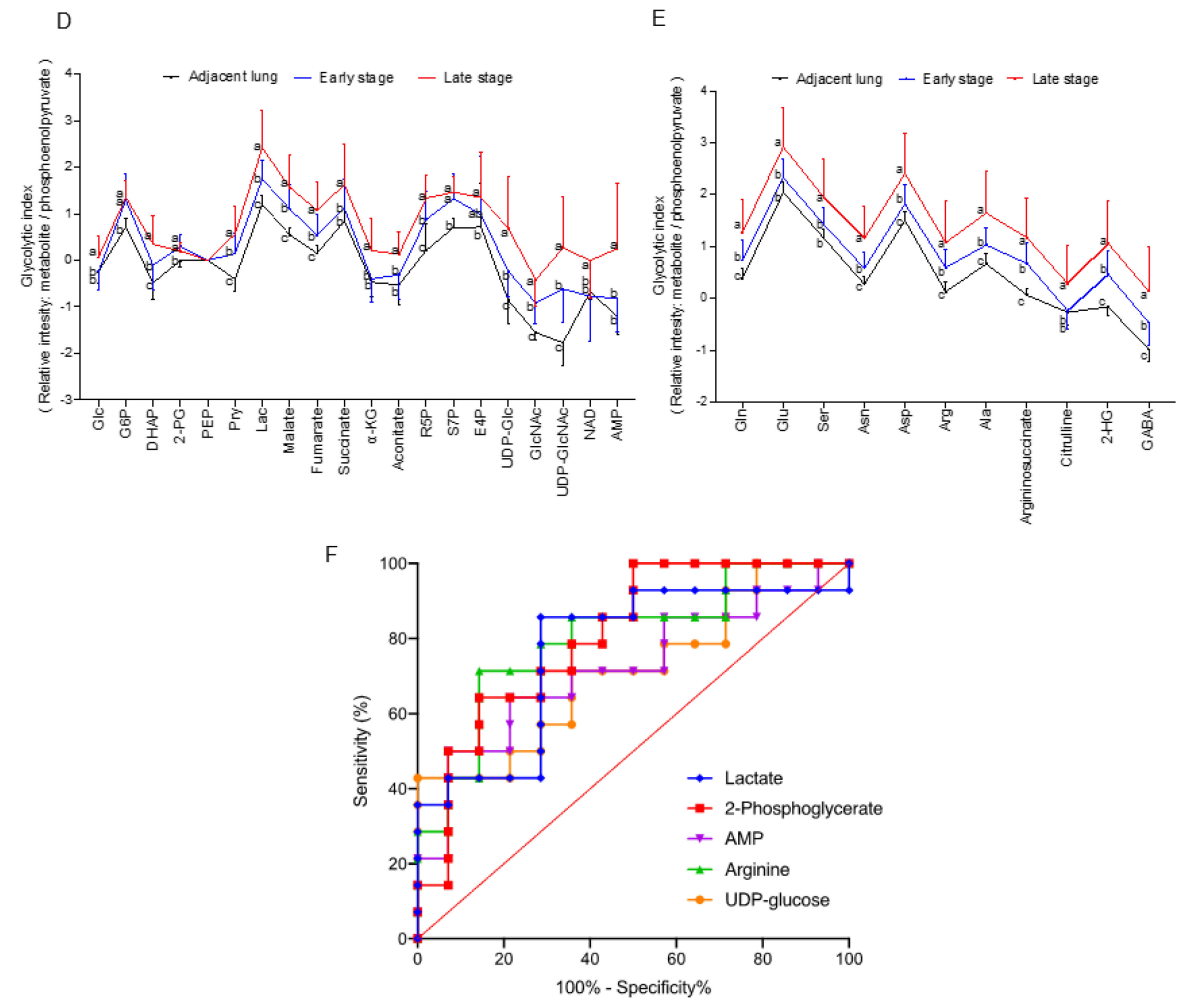
Discover stage-sensitive tumour metabolomics markers in paired NSCLCs (**A**) OPLS-DA of tissue samples for NSCLC and paired adjacent lungs; (**B**) VIP analysis on important metabolites that distinguish NSCLC from adjacent lungs; (**C**) Multivariate model of volcano plot analysis identifies significantly changed metabolites between tumours and paired lungs based on the selection criteria of VIP > 1, *p* < 0.05, R < 0.05, and fold-change > 2. Glycolytic index of metabolites (metabolites/PEP ratio) in (**D**) lactate metabolism pathways and (**E**) gluconeogenic amino acids of NSCLCs. Variables without common letter differed at *p* < 0.05. (**F**) ROC curve of each potential stage-sensitive biomarkers in paired NSCLCs. The ROC curve was plotted using GraphPad Prism 9.

Metabolomics data in the paired NSCLCs were analysed by MetaboAnalyst 5.0 for multivariate analysis and normalized by sum normalization, cube root transformation, and auto scaling. OPLS-DA was first used to explore the metabolites disparities between the NSCLC and paired lungs. As illustrated in Figure 1A, a clear trend of separation manifested significant metabolic alterations in tumours and paired lungs. OPLS-DA was used as the supervised model to predict the different changes by a 10-fold cross-validation (CV) and 1000-times permutation test (*p* < 0.001) in paired NSCLCs. After OPLS-DA, VIP analysis was applied to identify 15 important metabolites that contribute to classification (Figure 1B). Among those 15 important metabolites, lactate, glucose, and N-acetylglucosamine (GlcNAc) rank as the top three tumour markers to contribute metabolic diversity in NSCLCs. A volcano map was drawn to show how the 15 metabolites changed significantly (Figure 1C). According to FDR < 0.05 and log_2_FC > 2, of which 10 metabolites (lactate, GlcNAc, arginosuccinate, pyruvate, malate, UDP-N-acetylglucosamine (UDP-GlcNAc), sedoheptulose-7-phosphate (S7P), 2-hydroxyglutarate, UDP-Glucose, and AMP) were significantly up-regulated, and one of phosphoenolpyruvate (PEP) was down regulated in NSCLCs. The tumour markers were then normalized with PEP (glycolytic flux intermediate) to express the glycolytic index. As compared with the early stage-tumours and the paired adjacent lungs, advance tumours displayed higher glycolytic indexes for metabolites in lactate metabolism (glucose, pyruvate, and lactate), TCA cycle (malate, fumurate, succinate, α-ketoglutarate, and aconite), pentose phosphate pathway (PPP) (ribose-5-phosphate (R5P), erythrose-4-phosphate (E4P), (S7P), nucleotide biosynthesis of AMP, and amino sugar metabolism (UDP-Glucose, GlcNAc, and UDP-GlcNAc) (Figure 1D). In particular, contents of gluconeogenesis amino acids (glutamine, glutamate, aspartate, asparagine, arginine, alanine, and serine) were significantly enriched in advance tumours (Figure 1E). The area under the curve (AUC) of the ROC analysis was applied to evaluate the classification performance of the above stage-sensitive tumour markers by GraphPad prism 9. Among those potential tumour biomarkers, lactate (AUC = 0.765, Sig = 0.017, CI: 0.580–0.951), 2-phosphoglycerate (AUC = 0.806, Sig = 0.005, CI 0.643–0.969), AMP (AUC = 0.719, Sig = 0.048, CI 0.525–0.914), arginine (AUC = 0.801, Sig = 0.006, CI 0.634–0.968), and UDP-glucose (AUC = 0.714, Sig = 0.053, CI 0.522–0.907) displayed the high accuracy in diagnosing advance NSCLC (Figure 1F). 

### 3.2. Association of Clinical Folate Trait with Advance Stage-Sensitive Tumour Markers in NSCLCs

**Figure 2:**
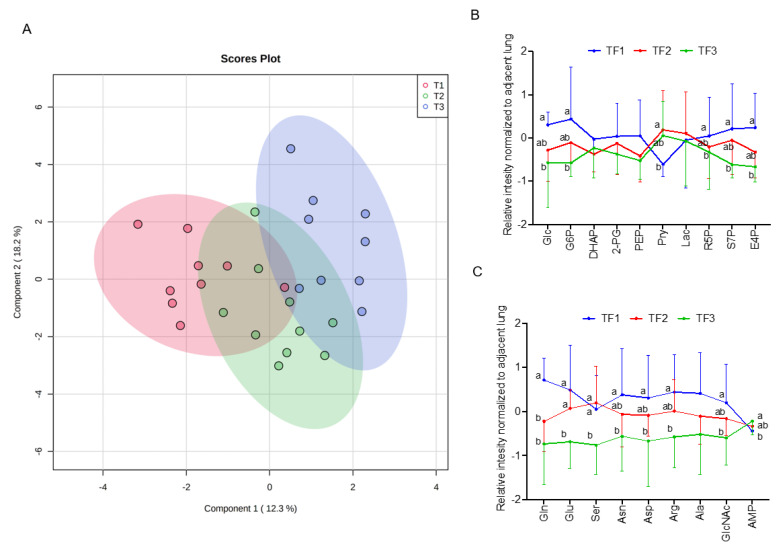

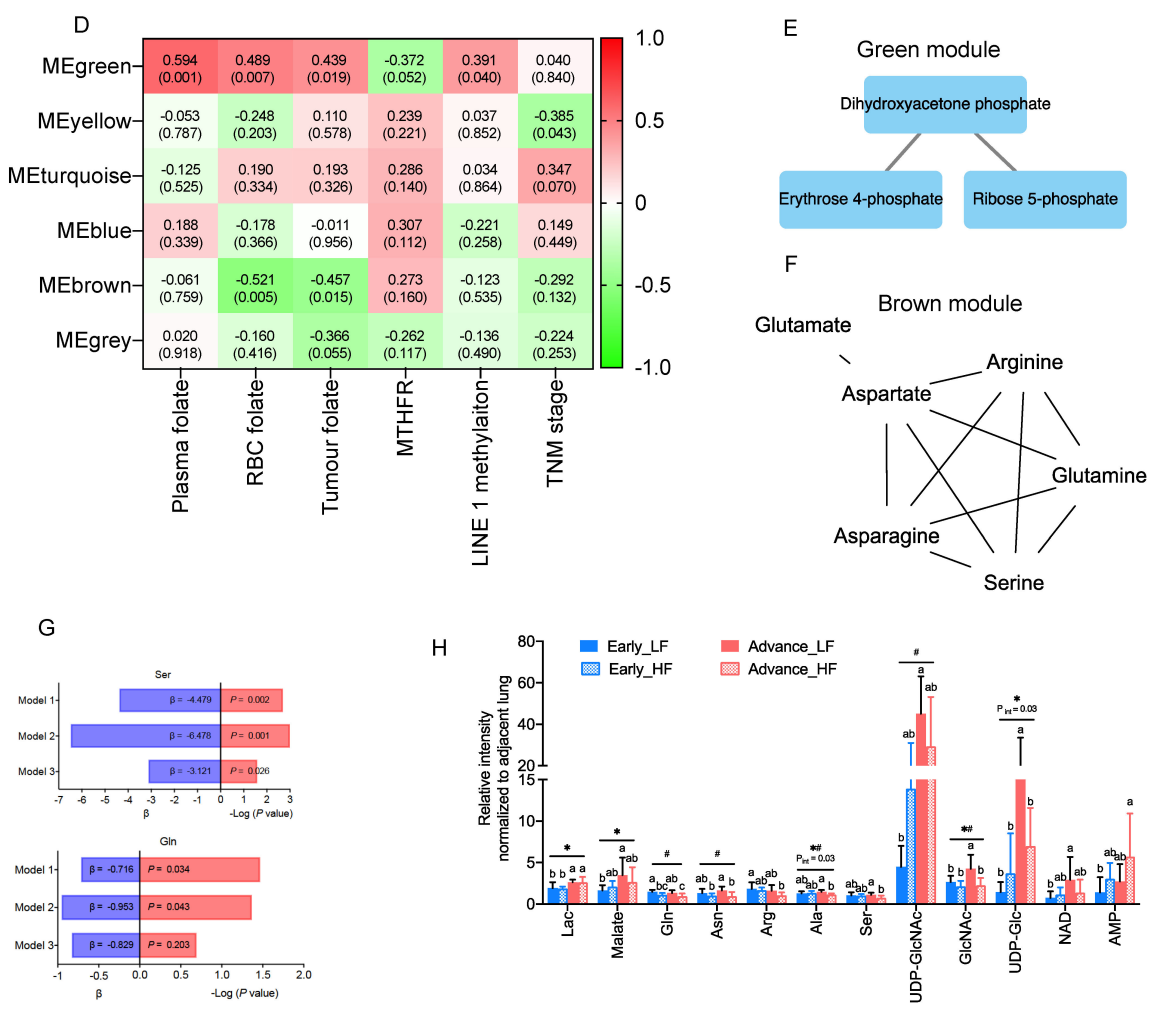
Association of clinical folate trait with stage-sensitive tumour markers in NSCLCs. (**A**) OPLS-DA of paired NSCLCs stratified by tertile tumour folate. Relative abundance of metabolites in (**B**) central carbon and (**C**) glycolytic amino acids metabolisms among tertile tumour folate-stratified NSCLCs. Signal intensity of the designated tumour metabolites was normalized with each paired adjacent lung. Z-scored values were expressed as mean ± SD. (**D**) Identify metabolite modulates associated with clinical folate traits by weighted gene co-expression network analysis (WGCNA). Each row stands for a module metabolite (MM), and each column represents a clinical folate marker. Each long square contains the correlation coefficient with *p* value in parenthesis. Differential enriched metabolites network in the clinical folate-associated (**E**) green and (**F**) brown module. Red block represents the hub metabolites. (**G**) Multivariable linear regression models were constructed to analyse the association of tumour folate with glutamine and serine. Model 1: adjusted for age, sex, smoking status, and BMI. Model 2: additional adjustment for plasma folate; Model 3: additional adjustment for genomic epigenetic mark (*LINE1* methylation) and genetic polymorphism of *MTHFR 677CT/TT* genotypes. (**H**) Interaction of tumour folate and TNM stage analysed by two-way ANOVA. NSCLCs were stratified into LF and HF groups by median cut out levels at early (IA) and advance stage (IB-IV). Data were expressed as the ratio of tumour metabolites normalized with those of adjacent lungs. Variables without common letter differed at *p* < 0.05. * *p* < 0.05 between early and advance stage. ^#^ *p* < 0.05 compared between the LF and HF group. *p* for interaction < 0.05 compared between tumour folate and TNM stage.

Next, we explore association of clinical folate trait with advance stage-sensitive tumour metabolomics markers. As shown in Figure 2A, the clear trend of separation manifested the existence of significant metabolic alterations with tertile tumour folate. Low (LF: TF1) vs. TF2 and TF3 tumours displayed significantly higher metabolite content in glycolysis pathway (glucose and glucose-6-phosphate), PPP pathway (R5P, S7P, and E4P) (Figure 2B), and glycogenic amino acids (glutamine, glutamate, asparagine, aspartate, arginine, serine, and alanine) (Figure 2C). T1 vs. T2/T3 folate was associated with higher glycolytic index for lactate, malate, glutamine, and its intermediate partners compared with HF, and adjacent lungs in advance stage (Figure S2). By WGCNA analysis to identify metabolite module and its correlation with systematic folate traits, six modules were obtained, of which the green module clustered 3 tumour metabolites (dihydroxyacetone phosphate, E4P, and R5P) associated with change of plasma folate (r = 0.594, *p* = 0.001), RBC folate (r = 0.489, *p* = 0.007), tumour folate (r = 0.439, *p* = 0.019), and genomic DNA methylation (r = 0.391, *p* = 0.04) (Figure 2D,E). The brown module clustered six tumour metabolites (glutamine, serine, aspartate, asparagine, arginine, and glutamate), which were inversely associated with changes of RBC folate (r = −0.521, *p* = 0.005), and tumour folate (r = −0.457, *p* = 0.015) (Figure 2D,F). Serine and asparagine (red block) were identified as the hub metabolites navigating the contents of glutamine, glutamate, and aspartate in the network analysis (Figure 2F). Tumour folate predicted altered tumour serine (Model 3: beta: −3.12, *p* = 0.026) and glutamine (Model 2: beta: −0.95, *p* = 0.043) after multivariable adjustment of age, sex, smoking status, BMI, plasma folate, genomic *LINE1* methylation, and *MTHFR 677CT/TT* genotype (Figure 2G). Two-way ANOVO analysis revealed that TF1 (low folate: LF) vs. TF3 (high folate: HF) was significantly associated with elevated metabolites in the brown module (glutamine, aspartate, serine, and alanine) only for advance tumours (Figure 2F,H). No stage × tumour folate interaction effect was detected except for alanine (*p* for interaction = 0.03) (Figure 2H). 

### 3.3. Enrichment Analysis on Metabolic Pathways by Folate-Responsive and Stage-Sensitive Tumour Metabolomics Markers

**Figure 3:**
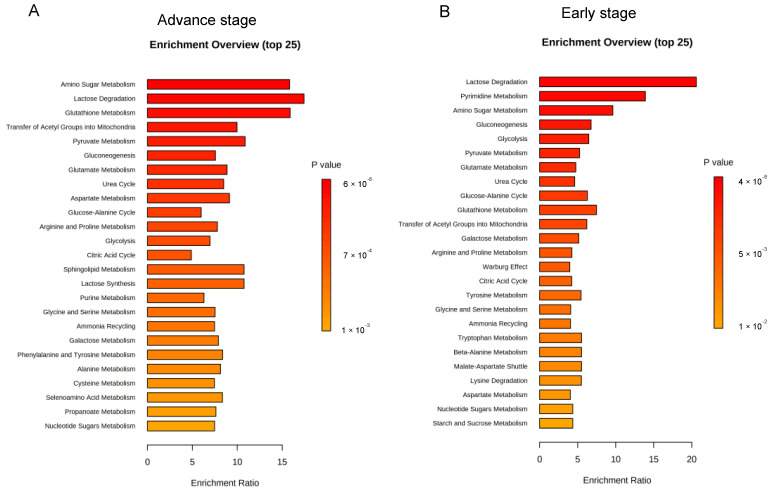
Enrichment analysis on metabolic pathways by folate-responsive and stage-sensitive tumour metabolomics markers. Enrichment analysis based on KEGG (Kyoto Encyclopaedia of Genes and Genomes) of the differential metabolites between suboptimal folate-NSCLC and paired lung tissues using MetaboAnalyst 5.0 (http://www.metaboanalyst.ca/, accessed on 21 December 2021). Enrichment metabolic pathways were analysed for the metabolomics signatures of low folate-tumours at (**A**) early stage and (**B**) advance stage. Median tumour folate was the cut out to stratify low and high folate-exposed NSCLCs. *p* ≤ 0.05 was used to select the significant enrichment. Bar length presents for enrichment ratio. Colour intensity of scale bar represents for log *p* values.

To obtain biological information related to the overrepresentation function of the identified folate-responsive tumour markers as to cancer stage, enrichment analyses were performed. For advance tumours with LF status, the “amino sugar metabolism” ranks the top one enriched pathway, followed by “glutathione metabolism”, “transfer of acetyl group into mitochondria”, “pyruvate metabolism”, “gluconeogenesis”, “glucogenic amino acid metabolism”, “glucose-alanine cycle”, and “urea cycle” (Figure 3A). Differential metabolic enrichment profiles were detected for early stage-tumours with LF status (Figure 3B). The “lactose degradation” and “pyrimidine metabolism” ranked the top two enriched pathways, respectively, with moderately enriched Warburg effect pathway. 

### 3.4. The Transcriptomics Signatures Associated with Folate-Sensitive and Stage-Responsive Tumour Metabolomics Markers 

**Figure 4:**
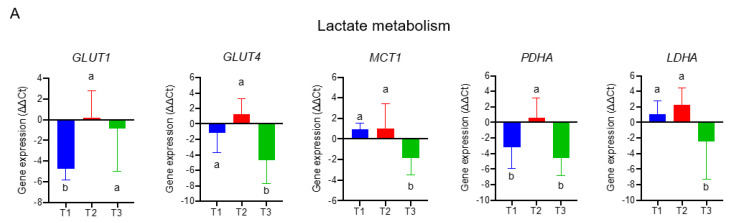

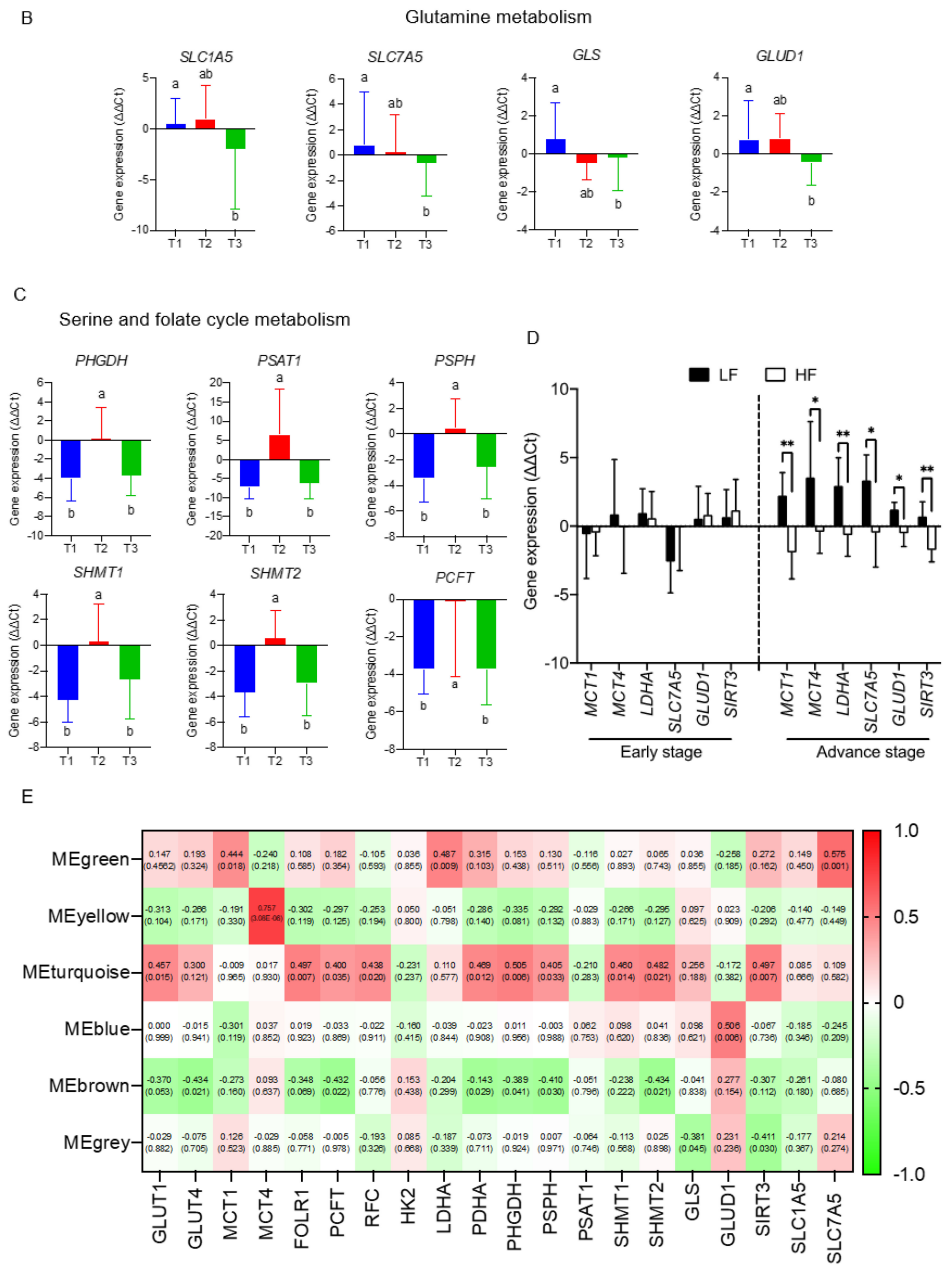

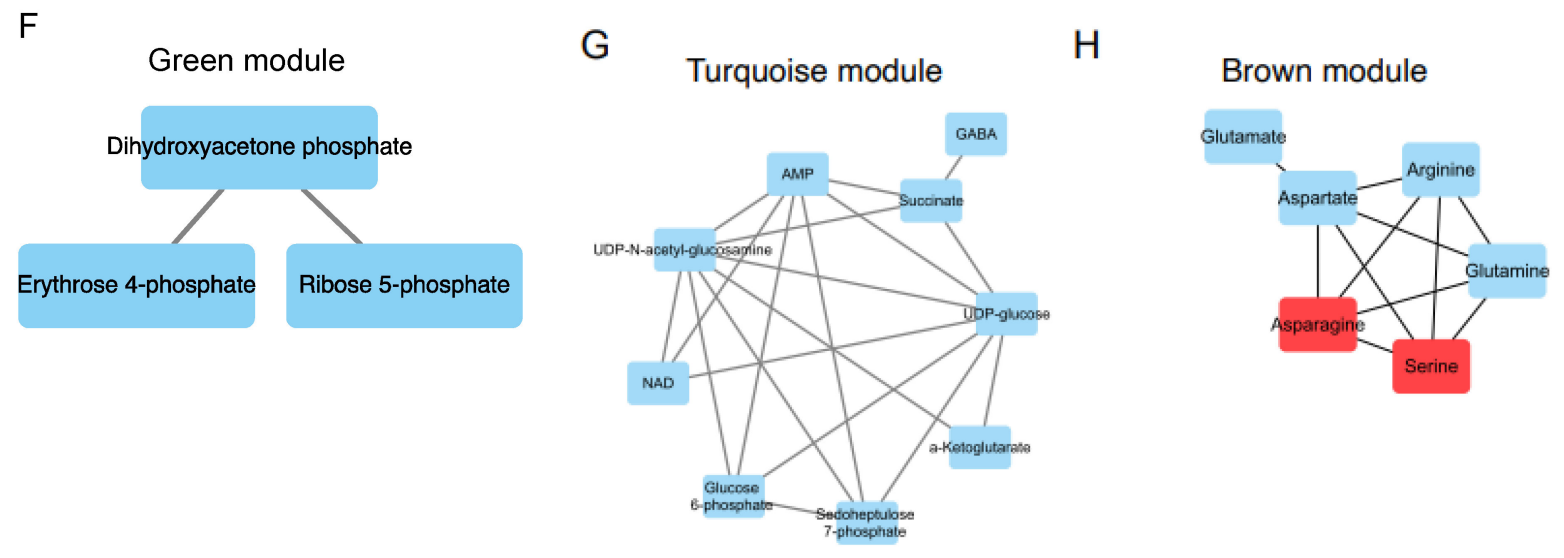
The transcriptomics signatures associated with folate-sensitive and stage-responsive tumour metabolomics markers. By tertile tumour folate stratification, transcript levels of regulatory enzymes and nutrient transporters involving (**A**) lactate metabolism, (**B**) glutamine metabolism, and (**C**) serine and folate cycle metabolism in pair NSCLCs were analysed by qPCR. Glyceraldehyde 3-phosphate dehydrogenase (GAPDH) was used as reference gene. Variables without common letter differed at *p* < 0.05. (**D**) Differential expression gene (DEGs) stratified by advance stage and altered tumour folate. Data are presented as delta delta Ct for relative expression as opposed to the paired lungs. Means for variables without common letter differed at *p* < 0.05 by One-way ANOVA (analysis of covariance) following a Duncan test or a student *t* test. * *p* < 0.05; ** *p* < 0.01. (**E**) Metabolite modulates associated with DEGs clustering by weighted gene co-expression network analysis (WGCNA). Each row stands for a module metabolite (MM), and each column represents DEGs. Each long square contains the correlation coefficient with *p* value in parenthesis. Differential enriched metabolites network in folate-associated (**F**) green, (**G**) turquoise, and (**H**) brown module. Red block represents the hub metabolites.

To delineate molecular mechanisms for folate-responsive and stage-sensitive tumour metabolomics markers, we performed a transcriptomics analysis targeting the metabolic genes of regulatory enzymes and nutrient transporters in one-carbon and glycogenic amino acids metabolism (Figure 4). Compared with the T2 tumours (control), transcript levels of glucose transporters (*GLUT1* and *GLUT4*) and pruvate dehydrogenase A (*PDHA*)*,* the rate-limiting enzyme for mitochondria bioenergetic, were differentially up-regulated in T1 and T3 tumours. Both T1 and T3 tumours overexpressed the monocarboxylic transporter 4 (*MCT4*) (data not shown) for lactate export, whereas T3 vs. T1 tumours specifically up regulated transcript expression of monocarboxylic transporter 1 (*MCT1*) for lactate import and lactate dehydrogenase A (*LDHA*) for lactate metabolism (Figure 4A). Expressions of four transcripts including L-type amino acid transporter 1 (*SLC7A5*), the high-affinity L-glutamine transporter (*SLC1A5*), glutaminase (*GLS*), and glutamate dehydrogenase 1 (*GLUD1*) were all significantly up regulated in T3 as compared with T1 and T2 tumours (Figure 4B). Transcripts expressions of three key enzymes (phosphoglycerate dehydrogenase (*PHGDH*), phosphoserine phosphatase (*PSPH*), and phosphoserine aminotransferase (*PSAT1*)) involving in de novo serine synthesis, and two key enzymes that channel serine into folate-cycle as the cytosolic serine hydroxymethyltransferase 1 (*SHMT1*) and mitochondrial *SHMT2*, and the proton-coupled folate transporter (*PCFT*) are highly over expressed in T1 and T3 vs. T2 tumours (Figure 4C). The above differential expression genes (DEGs) in response to altered tumour folate were TNM stage sensitive. Only for advance NSCLC, high vs. low tumour folate was significantly associated with the overexpression of *MCT1*, *MCT4*, *LDHA*, *SLC7A5*, *SIRT3*, and *GLUD1* (Figure 4D). When these folate-responsive DEGs were selected for WGCNA, six modules of metabolites were obtained, of which the MEgreen, turquoise, and brown modules clustered the metabolites most sensitive to DEGs (Figure 4E). In MEgreen module, increased expression of *MCT1* (r = 0.444, *p* = 0.018), *LDHA* (r = 0.487, *p* = 0.009), and *SLC7A5* (r = 0.575, *p* = 0.001) was significantly associated with enriched metabolites in glycolytic and PPP (dihydroxyacetone, E4P and R5P) (Figure 4 E,F). In MEturquoise module, increased expression of *GLUT1* (r = 0.457, *p* = 0.015), *FOLR1* (r = 0.497, *p* = 0.007), *PCFT* (r = 0.4, *p* = 0.035), *RFC* (r = 0.438, *p* = 0.02), *PDHA* (r = 0.469, *p* = 0.012), *PHGDH* (r = 0.505, *p* = 0.006), *PSPH* (r = 0.405, *p* = 0.033), *SHMT1*(r = 0.46, *p* = 0.014), *SHMT2* (r = 0.482, *p* = 0.021), and *SIRT3* (r = 0.497, *p* = 0.007) were associated with nine enriched metabolites in PPP (glucose-6-P and S7P), TCA cycle (succinate, α-ketoglutarate and GABA), nucleotide synthesis (AMP and NAD), and amino sugar (UDP-glucose and UDP-GlcNAc) metabolic pathways (Figure 4E,G). In MEbrown module, increased expression of *GLUT4* (r = −0.434, *p* = 0.021), *PCFT* (r = −0.432, *p* = 0.022), *PDHA* (r = −0.143, *p* = 0.029), *PHGDH* (r = −0.389, *p* = 0.041), *PSPH* (r = −0.410, *p* = 0.03), and *SHMT2* (r = −0.434, *p* = 0.021) was inversely associated with decreased content of nine glycolytic amino acids metabolism including glutamine, serine, arginine, aspartate, asparagine, and glutamate (Figure 4E,H). 

### 3.5. The Interactive Network of Metabolites and Genetic Signatures Modified by Advance Stage and Clinical Folate Trait in NSCLCs 

**Figure 5:**
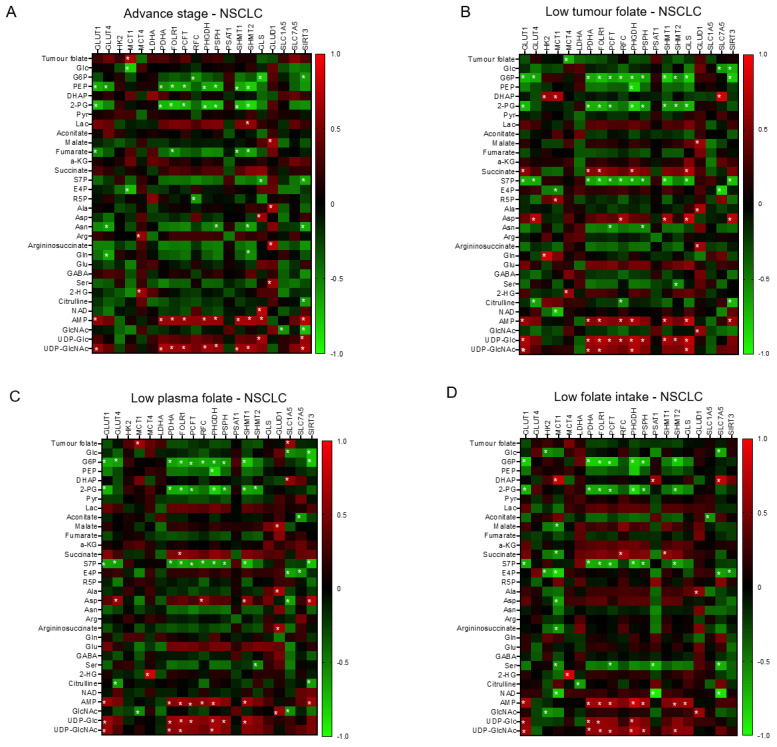

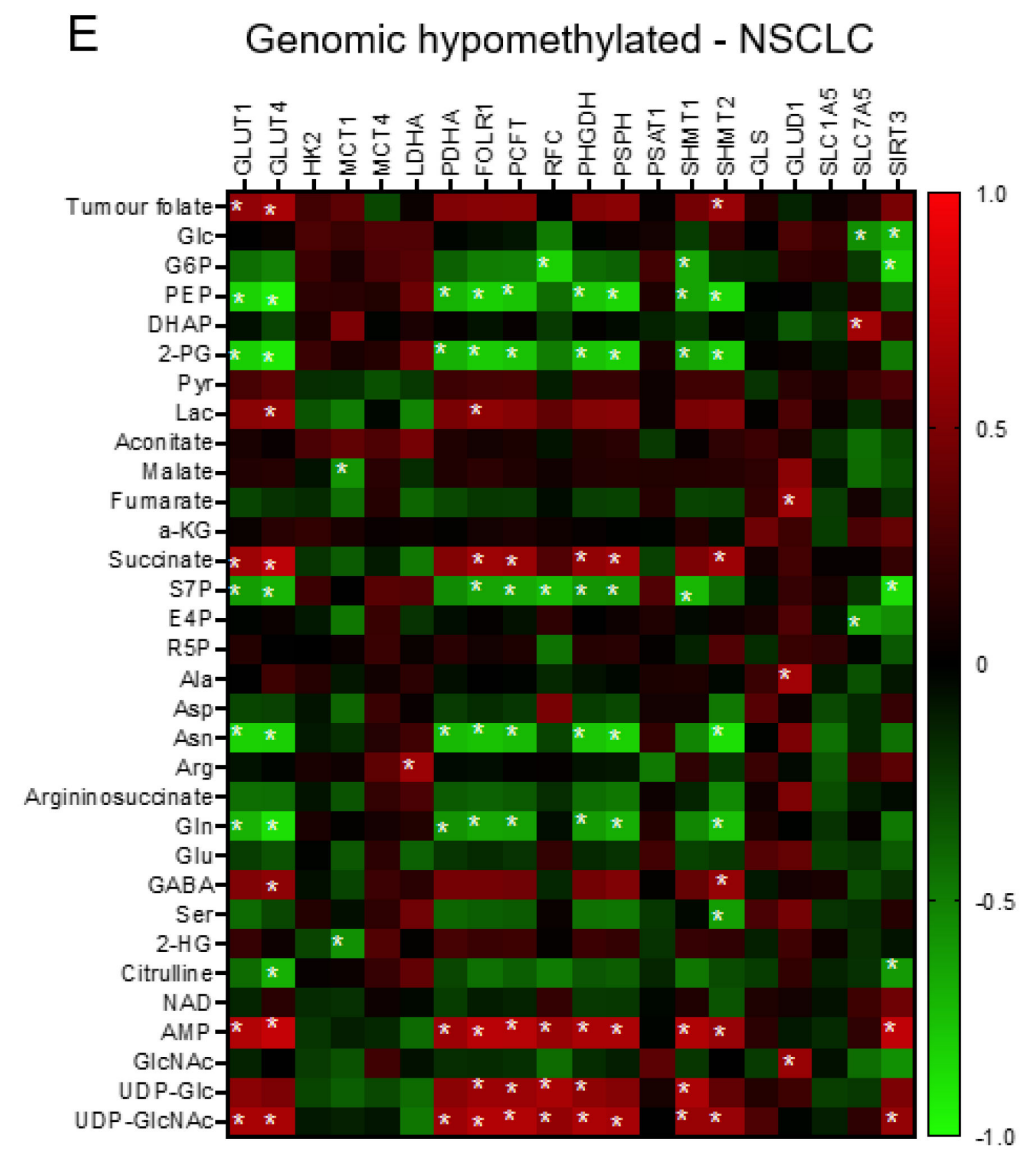
The interactive network of metabolites and genetic signatures modified by advance stage and clinical folate trait in NSCLCs. The correlation heat map was constructed for (**A**) advance stage-NSCLC; (**B**) low tumour folate-NSCLC (< media tumour folate); (**C**) low plasma folate-NSCLC (< median blood folate: 5.5 ug/mL); (**D**) low dietary folate intake-NSCLC (media intake: 497 ug/day); and (**E**) genomic DNA hypomethylation (*LINE1* methylation < 66%). The Pearson coefficient correlation was considered to be statistically significant at * *p* < 0.05.

We construct the partial correlation heat map and interactive network for metabolites and genetic signatures in responsive to advance stage and altered clinical folate trait. For aggressive tumours (Figure 5A), decreased tumour folate was strongly associated with elevated serine, glutamine, and glucose, and weakly correlated with AMP as illustrated in the interactive network (Figure S5). Elevated glutamine was specifically associated with decreased expression of *GLUT4* and *SHMT2*, and elevated serine with increased *GLUD1* expression (Figure 5A). Elevated aggressive tumour marker of AMP and UDP-GluNAc was associated with up regulated transcript expression of clustering metabolic enzymes involving in glucose transport (*GLUT1*), mitochondria bioenergetics (*PDHA*), glutaminolysis (*GLS*), serine biosynthesis (*PHGDH* and *PSPH*), folate transport (*PCFT* and *FOLR1*), and folate cycle (*SHMT1* and *SHMT2*) (Figure 5A). These metabolites and gene interactive network signatures were not expressed in early stage-NSCLCs (Figure S4). NSCLCs with low tumour folate (<median: 138 ng/g) (Figure 5B), low plasma folate (<5.5 ng/mL; Figure 5C), and low dietary folate intake (<497 ug/day; Figure 5D) displayed onco-metabolite and genetic interactive network signatures resembling the aggressive tumours signature profile (Figure 5A) and the genomic hypomethylated-NSCLCs signature profile (Figure 5E). Such metabolite and genetic integrated network signatures did not express in the elevated folate trait counterparts (Figure S4). 

### 3.6. Folate-Responsive Tumour Signatures Predicted Overall Survival of Patients with Lung Cancers

**Figure 6:**
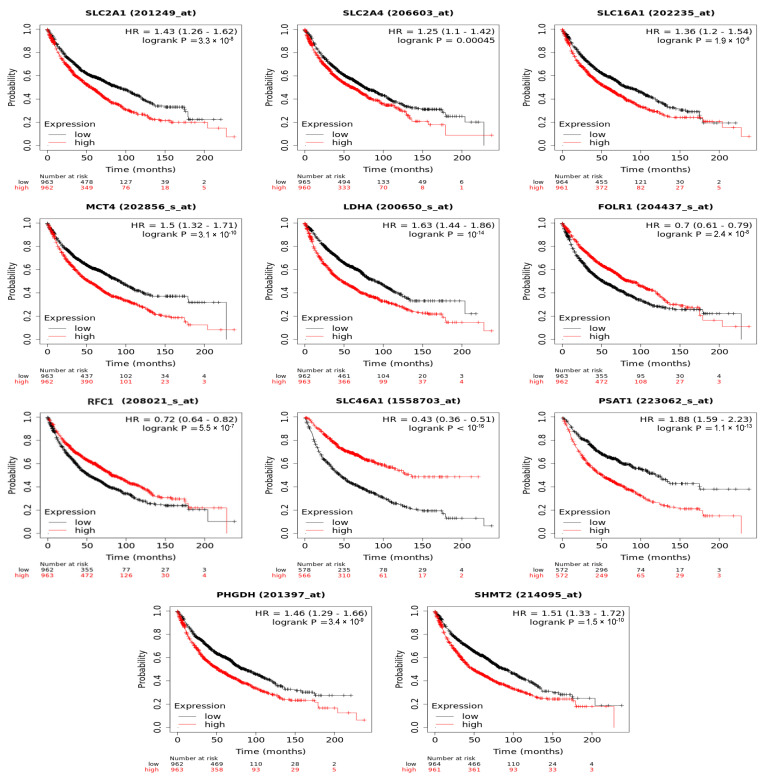
Folate-responsive aggressive tumours’ signatures predicted overall survival of patients with lung cancers. Kaplan–Meier survival curves for *ADSL* and *ATIC* genetic expression (high and low levels) was explored.

To demonstrate the clinical relevance of the folate-responsive and stage-sensitive tumour signatures, we explored the Kaplan–Meier survival curves to predict overall survival of LC patients. Higher expression of metabolic genes in glycolytic lactate and serine/folate cycle metabolism, such as *GLUT1* (HR = 1.43, log-rank *p* = 3.3 × 10^−8^), *GLUT4* (HR = 1.25, log-rank *p* = 0.00045), *MCT1* (HR: 1.36, log-rank *p* = 1.9 × 10^−6^), *MCT4* (HR = 1.5, log-rank *p* = 3.1 × 10^−10^), *LDHA* (HR: 1.63, log-rank *p* = 4 × 10^−14^), *PHGDH* (HR = 1.46, log-rank *p* = 3.4 × 10^−9^), *PSAT1* (HR = 1.88, log-rank *p* = 1.1 × 10^−13^), and *SHMT2* (HR = 1.51, log-rank *p* = 1.5 × 10^−10^), predicted poorer prognoses and lower diseases-free survival rates in LC patients. Higher expression of folate transporters, including *FOLR1* (HR = 0.7, log-rank *p* = 2.4 × 10^−8^), *RFC2* (HR = 0.72, log-rank *p* = 5.5 × 10^−7^), and *PCFT* (HR = 0.43, log-rank *p* < 1 × 10^−16^), predicted better prognoses and higher diseases-free survival rates (Figure 6).

## 4. Discussion

This is the first LC/MS/MS-based metabolomic analysis on systematic folate-modeling tumour metabolic perturbation in an East Asian NSCLC cohort. LF tumours vs. adjacent lungs displayed significantly higher glycolytic index of lactate and lactate-metabolizing intermediates in TCA, whereas tumour lactate predicted advanced stage of NSCLC patients. The metabolomics-identified tumour lactate marker is in line with enhanced glycolytic lactate production of human NSCLC based on in situ glucose flux analysis [22,23,39,40]. By ^13^C-lactate trace labelling, Faubert et al. [18] demonstrated that early-stage LC patients with high glycolytic index of lactate/3-phosphoglycerate progressed to distant metastases after several years of surgery. The lactate-dependent anabolism was reported to be part of aggressive NSCLC with the prognostic prediction in NSCLC patients [19,41,42,43]. Increased lactate coupling with elevated purine metabolites in adenocarcinoma lung tissue sustains cancer proliferation and fast progression [44,45,46]. As indicated in the animal study, folate deprivation of lung carcinoma-transplanted mice promoted hyperglycolytic lactate production in lungs to enhance lung cancer metastasis [8]. Our findings, together with results of the other studies, unveil the aggressive NSCLC marker of lactate in response to tumour folate depletion, which proposed a new metabolic link plausible to explain the association of inferior folate status with LC malignancy from clinical and epidemiologic studies [14,15]. 

The novel finding in the present study is to identify glutamine and its intermediate partners (glutamate, aspartate, arginine, and asparagine) as the distinctive LF-responsive and stage-sensitive tumour signatures. Decreased tumour folate predicted the elevated magnitude of tumour glutamine in aggressive but not in early staged-NSCLCs after multiple variables adjustment (β = −0.77, *p* = 0.01). WGCNA revealed the clustering metabolite module of glutamine and its intermediate partners associated with inferior RBC and tumour folate of NSCLCs. When grown in folate-deprived media, the invasive and metastatic breast cancers displayed highly elevated glutamine-associated glucogenic amino acids (glutamate, aspartate, and asparagine) [47]. In line with the In vivo evidence, the altered glutamine biosynthesis defined sensitivity of lung metastasis [48,49], yet the mechanistic link of tumour LF with elevated glutamine signature as to NSCLC malignancy remains obscure. Several hypotheses are plausible. It has been demonstrated that lung tumours synthesize glutamine from glucose-derived carbon [50]. We have observed that LF tumours up regulated transcript expression of glucose transporter 1 to increase glucose uptake, which was significantly correlated with enriched tumour glutamine as shown in the partial correlation heat map. Given that LF tumours displayed high glycolytic index of glutamine specific to aggressive cancer stage, the data suggest that LF promoted glucose-derived glutamine production by transcriptional regulation of targeting glucose metabolism. On the other hand, LF tumours displayed suppressed expression of *GLS* and *GLUD1,* referring a decreased glutaminolysis to enter into TCA cycle for mitochondria oxidative bioenergetics. Preserved glutamine of LF tumours may favour glutamine-engaging metabolic pathways in ammonia recycling, nucleotide synthesis, and amino-sugar metabolism [49,51]. Indeed, amino sugar metabolism ranked the top one enriched biochemical pathway in LF tumours at advance stage rather than at early stage. Furthermore, glucose flux analysis revealed that glutamine serves as the important precursor for glutathione synthesis, a key cellular antioxidant, critical for redox homeostasis and lung cancers progression [52,53]. Glutamine elevation to maintain redox homeostasis could be the prioritized metabolic choice of LF tumours under oxidative stress [54,55] for survival from oxidative signalling-apoptosis [9] and for malignant LC progression [53]. In line with the metabolic readout for LF-modifying glutamine-metabolizing genetic expression, glutathione metabolism ranked in the top two enriched pathway in LF but not in HF tumours in a stage-dependent manner. Collectively, our data suggested that LF modified transcriptomics expression to minimize glutaminolysis and promote glucose-derived glutamine synthesis, which supports amino sugar, nucleotide metabolism, and redox homeostasis rather than mitochondrial bioenergetics metabolism [50]. The causal malignancy relationship between low folate and glutamine marker warrants confirmation studies.

Another key finding in the present study is that serine—a proteinogenic amino acid and the source of folate 1C units [1]—acts as a hub metabolite navigating the metabolic network of glutamine and its intermediate amino acids identified by WGCNA. In our cohort, decreased tumour folate levels were associated with increased serine levels in an advance stage-dependent manner. This is the first human NSCLC-derived data revealing a stage-sensitive relationship between low tumour folate and high serine—the oncometabolite of the therapeutic target for cancers [56,57,58]. How altered tumour folate reprogramed serine metabolism to support malignancy transformation remains unclear. It is well documented that cancers regulated three metabolic pathways to augment cancer serine content: (1) the glycolysis and glutaminolysis to provide 3-phosphoglycerate (3-PG) and glutamate, respectively, to fuel do novo serine synthesis pathways (SSP) through metabolic enzymes of PHGDH, PSAT1, and PSPH [5,59]; (2) increased take up of extracellular serine to augment intracellular serine through L-type amino acid transporter 1 (SLC7A5) [60,61]; and (3) production of serine from glycine through methyltransferases SHMT1 (cytoplasmic) and SHMT2 (mitochondrial) [62,63,64]. The WGCNA on DEFs of paired NSCLCs revealed association of low tumour folate with increased transcripts of *GLUT1/4*, *PHGDH*, *PSPH*, and *PSAT1* in NSCLCs, suggesting increased glucose uptake and enhanced glycolytic SSP pathways to contribute cancer serine levels under folate-deficit condition. In parallel, LF tumours expressed 2-fold higher SHMT1 and SHMT2 levels than did the paired adjacent lung tissue. Mitochondrial serine metabolism by SHMT2 enables cytosolic folate coenzymes for nucleotide synthesis and serine regeneration by SHMT1 [64,65]. The protective effect of SHMT1/2 overexpression includes cancer cell survival through redox maintenance and hypoxic stress reduction [1]. Without modifying transcript level of SLC7A5 for serine import, LF tumours increased transcript levels of proton-coupling folate transporter (PCFT) to enhance exogenous folate uptake under lactate-acidified microenvironment [66] and to compensate folate-deficit condition. After multivariable adjustment, decreased tumour folate predicted elevated tumour serine, independent of MTHFR genotypes and DNA methylation effect. The data suggest the additional mechanism by which LF mediated increased serine metabolism. Our WGCNA revealed that SIRT3 was inversely associated with MEBrown module for serine/glutamine-derived amino acid signatures (r = −0.047, *p* = 0.012). Low folate was associated with repressed transcript of SIRT3 in advance NSCLCs, suggesting the critical role of deacetylation on serine metabolism in low-folate NSCLCs, which warranted studies. 

It is notable that HF tumours displayed distinctive different tumour signatures from LF tumours. In multivariable adjusted models, up regulation of *LDHA* expression is associated with high tumour folate dose levels at advance NSCLC. LDHA is the key enzyme that catalyses the NADH-dependent reduction in pyruvate to lactate, a step essential for regenerating the NAD^+^, which is required for maintaining glycolysis and other metabolic activities. In highly glycolytic NSCLCs (accounting for >85% LCs) [67], LDHA overexpression is the key event for the enhancement of aerobic glycolysis, which promotes tumour malignant behaviour, and invasive ability through the activation of epithelial–mesenchymal transition in lung adenocarcinoma [68]. Higher blood LDHA levels are strongly correlated with shorter progression-free and overall survival in patients with advanced NSCLCs treated with immune checkpoint inhibitors [69]. Other differentially regulated metabolic targets by HF are MCTs, which are the 12-segment transmembrane proteins that symport protons with monocarboxylic acids, mainly lactate, and, to a lesser extent, pyruvate, ketone bodies, and branched-chain amino acids [42]. MCT1 (*SLC16A1*) is ubiquitously expressed and has high affinity for serum lactate as the cellular lactate importer, whereas MCT4 (*SLC16A3*) is strongly expressed in glycolytic cancer tissues for intracellular lactate export [70,71]. MCT1 overexpression in p53-null LC cells advances xenograft tumorigenicity and angiogenesis, and *MCT1* mRNA is enriched in LCs [72]. Human NSCLC cell lines and tissues overexpress MCT4, which is associated with poor NSCLC prognosis [72] and decreased overall survival in a wide variety of cancers [73]. As in other studies, the HF tumours demonstrated higher expression of *MCT1* and *MCT4*, which serve as malignant LC prognosis predictors, than did advanced stage LF tumours. The final metabolic target of the Warburg effect is *GLUT1*, which is also known as solute carrier family 2 A1 (SLC2A1); it is a uniporter protein encoded by *SLC2A2* in humans [74]. GLUT1 functions as a rate-limiting element critical for glucose transport to tumour cells; its overexpression predicts short disease-free and disease-specific survival in patients with LCs [75,76]. In lung adenocarcinomas, high *GLUT1* expression is associated with poor differentiation grade and positive lymph node at diagnosis [77]. We observed that LF but not HF tumours had significantly increased *GLUT1* expression in a folate-responsive and stag-sensitive manner. The differential Warburg effect associated ontogenetic expression in the LF and HF tumours may partly explain dual role of LF and HF in driving the oncometabolomic shift from the hyperglycolytic phenotype to the aggressive oncogenetic phenotype in most NSCLCs. 

Several factors affect how the results should be interpreted. First is the potential for tissue microheterogeneity at the sub-biopsy levels. Dissected tumour–normal tissue pairs may include stromal and vascular cells, which have diverse metabolic processes compared with NSCLC tissues. The commonly mutated oncogenes and tumour suppressor genes in NSCLCs include *KRAS*, *EGFR*, *PIK3CA*, *BRAF*, *STK*, and *TP53*; these common cancer metabolic markers [18] were not assessed in our cohort. These possibly mutated genotypes and molecular heterogeneity warrant further analysis so as to model folate-mediated metabolites changes. In addition, the number of tumours analysed in each group was small. This may have prevented the detection and analysis of mutation-specific signatures in response to tumour folate changes. Another potential limitation for the observed association of tumour folate with cancer metabolic change may be due to reverse causation. A prospective experimental study with a larger sample size is required to analyse the predictive power of the folate-responsive and stage-sensitive markers for early NSCLC diagnosis and prognosis prediction. 

In summary, our study identified the novel diagnostic tumour markers of malignancy NSCLCs in responsive to biological folate change at threshold cut out levels. The integrated metabolomics and transcriptomics data classified systematic low folate-modified metabolite and genetic interactive network signatures to predict poor survival of LC patents. The findings highlight new translational opportunities for dietary folate interventions, anti-folate drug development, and diagnostic markers discovery for better prognosis of NSCLC in folate precision medicine.

## Data Availability

The data presented in this study are available in Supplementary Materials here.

## Supplementary Materials

The following supporting information can be downloaded at: https://www.mdpi.com/article/10.3390/nu15010003/s1, Figure S1: Flow chart of experimental design; Figure S2: Glycolytic index of metabolites metabolite/phosphoenopyruvate ratio of targeted metabolites in the adjacent lungs and median tumour folate-stratified tumours at early and advance staged-NSCLC; Figure S3: Figure S3: WGCNA of DEGs in NSCLCs to determine soft threshold in scale-free topology network; Figure S4: The metabolites and genes interactive network was constructed for tumour pairs as a whole and in the subgroups defined by early stage, high tumour folate, high plasma folate, high folate intake, and genomic DNA hypermethylation; Table S1: Demographic, clinical folate, and pathologic data of the paired NSCLCs according to TNM stage.
