# Supplementary material for: Probing Folate-Responsive and Stage-Sensitive Metabolomics and Transcriptional Co-Expression Network Markers to Predict Prognosis of Non-Small Cell Lung Cancer Patients
Source: Nutrients. 2022 Dec 20;15(1):3. doi: 10.3390/nu15010003 (PMC9823804; doi:10.3390/nu15010003)
Supplement: Supplementary file 1 [file nutrients-15-00003-s001.zip › nutrients-2044069-supplementary.pdf]

Table S1. Demographic, clinical folate and pathologic data of the paired NSCLCs according to TNM stage.

Variables	Total paired lungs	Total tumours	TNM stage		*P* value
			IA	1B-IVB	
	n=28	n=28	n=14	n=14	
Age, year	61.8±8.0		62.1±7.9	61.4±8.6	0.838
Gender, n (%)					1.000
Male	4 (28.6%)		4 (28.6%)	4 (28.6%)	
Female	20 (71.4%)		10 (71.4%)	10 (71.4%)	
BMI, kg/m^2	23.1±2.6		22.3±2.4	23.9±2.6	0.102
Smoking status					0.360
Never smoking, n (%)	23 (82.2%)		12 (85.8%)	11 (78.6%)	
Former smoker, n (%)	4 (14.2%)		1 (7.1%)	3 (21.4%)	
Current smoker, n (%)	1 (3.6%)		1 (7.1%)	0 (0%)	
Dietary folate intake (mg/day)	597.36±347.00		482.27±269.26	712.43±386.08	0.080
Plasma folate (ng/mL)	8.27±4.38		8.36±4.33	8.19±4.60	0.924
RBC folate (ng/mL)	493.43±298.61		389.43±134.70	597.43±379.15	0.064
Lung tissue folate (ng/g)	265.38±219.09		300.65±244.36	230.11±193.13	0.405
Tumour tissue folate (ng/g)		205.12±160.84	174.99±110.22	235.24±199.07	0.333
Germ line MTHFR C677T polymorphism					0.225
CC, n (%)	9 (32.1%)		6 (42.9%)	3 (21.4%)	
CT/TT, n (%)	19 (67.9%)		8 (57.1%)	11 (78.6%)	

Lymphocytic LINE1 methylation , %	65.2±10.1	65.5±9.3	65.0±11.2	0.891
Tissue pathological characteristics, n (%)				
TNM				
IA	14 (50.0%)	14 (50.0%)	0 (0%)	0.000
IB, II, III, IV	14 (50.0%)	0 (0%)	14 (50.0%)	
T1	19 (67.9%)	14 (50.0%)	5 (35.7%)	0.000
Over T1	9 (22.1%)	0 (0%)	9 (64.3%)	
Histological grade, n (%)				0.482
G1	1 (3.6%)	1 (7.1%)	0 (0%)	
G2/G3	27 (96.4%)	13 (92.9%)	14 (50%)	

[1] Continuous variables are presented as mean and SD. Values between tumour stages were compared using Student's t-test. Discrete variable was expressed as numbers with proportions in parenthesis. Values between tumour stages were compared using the chi square test. Significance is defined at $P < 0.05$.

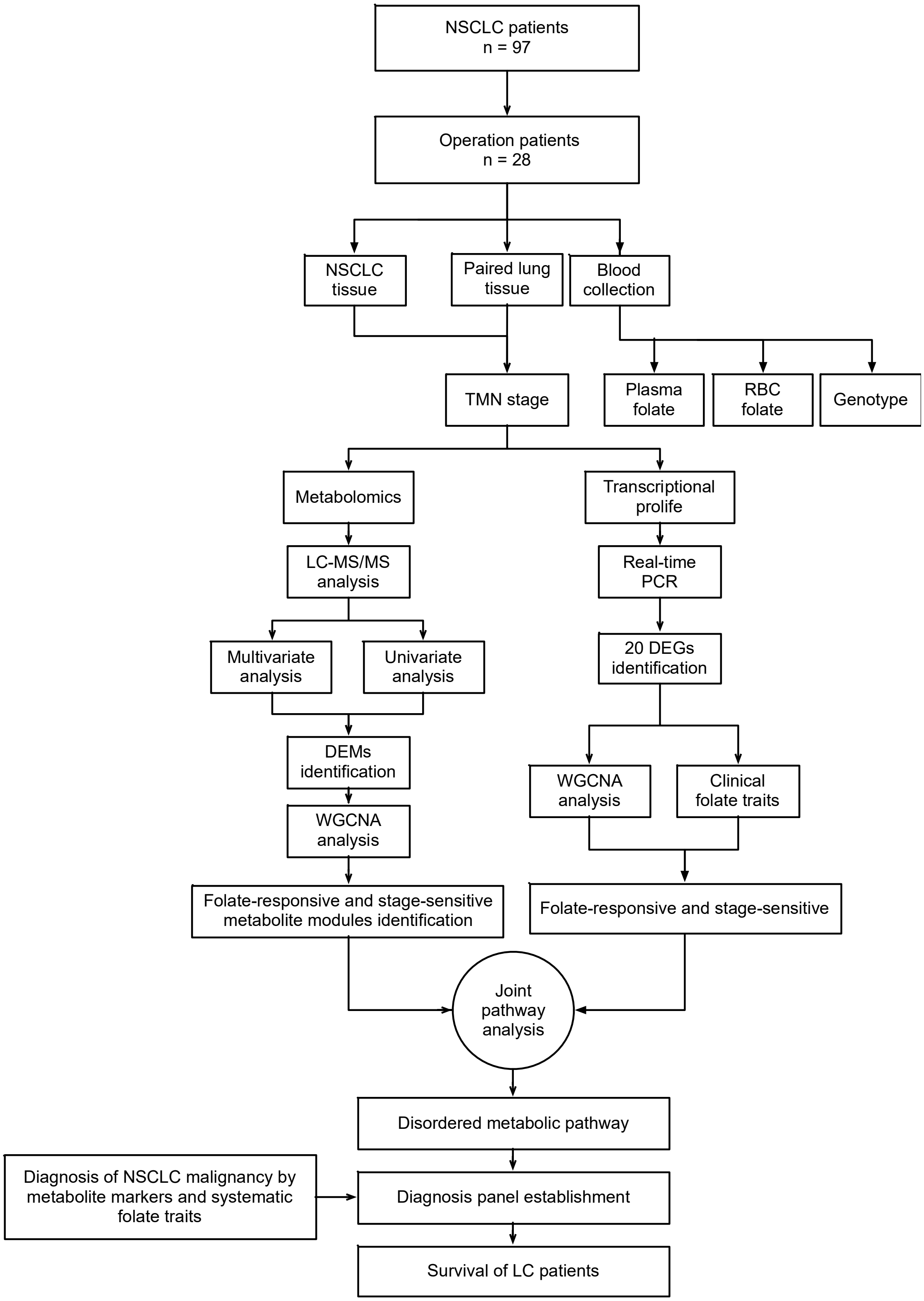

Figure S1. Flow chart of experimental design.

(A)

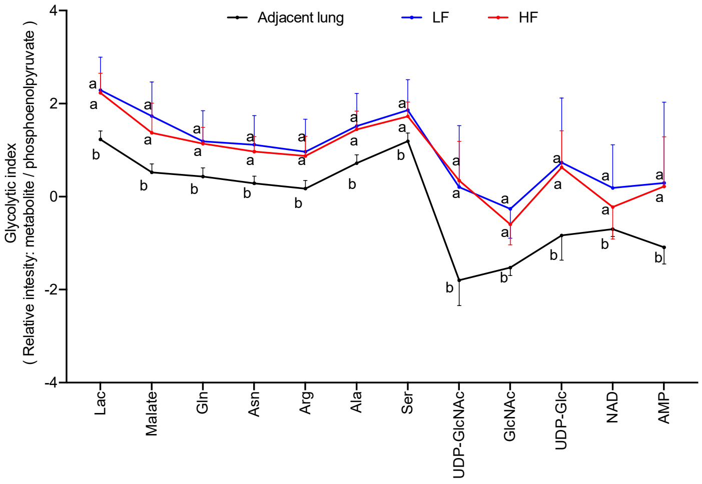

(B)

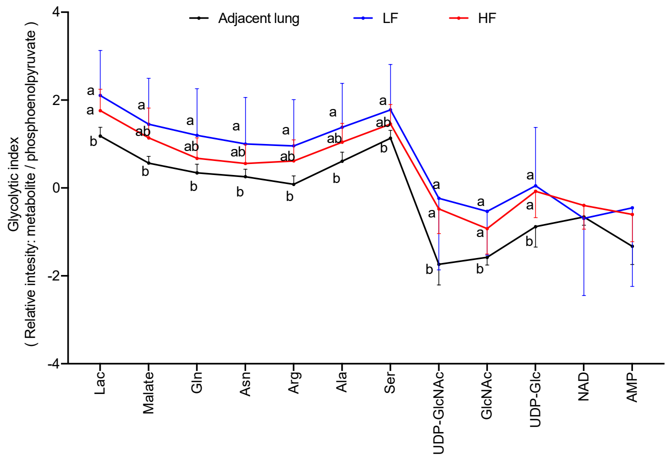

Figure S2. Glycolytic index of metabolites metabolite/phosphoenopyruvate ratio) of targeted metabolites in the adjacent lungs and median tumour folate-stratified tumours at early (A) and advance staged-NSCLC (B). Data were log transformed and expressed as mean ± SD (n = 28 for adjacent lungs, 14 for early stage-tumours, and 14 for advance-staged tumours).

(A)

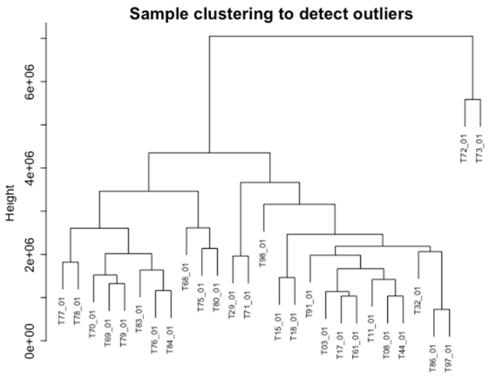

(B)

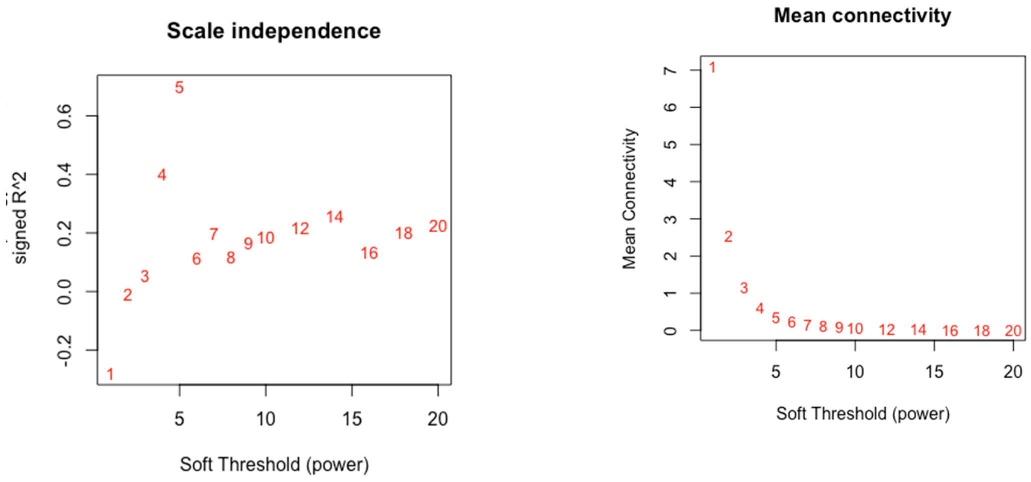

(C)

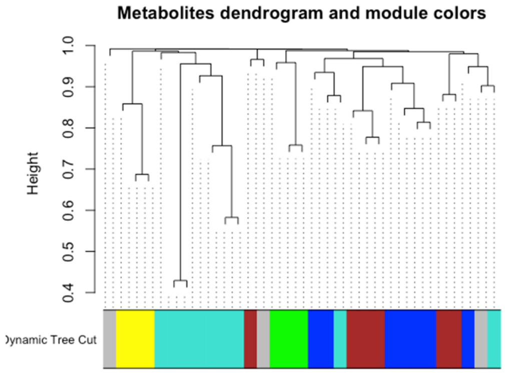

Figure S3. WGCNA of DEGs in NSCLCs to determine soft threshold in scale-free topology network**.** (A) Hierarchical clustering dendrogram of the sample. (B) Soft threshold selection process to obtain the scale-free fit index of network topology. (C) Module cluster tree showed seven key module eigengenes.

(A)

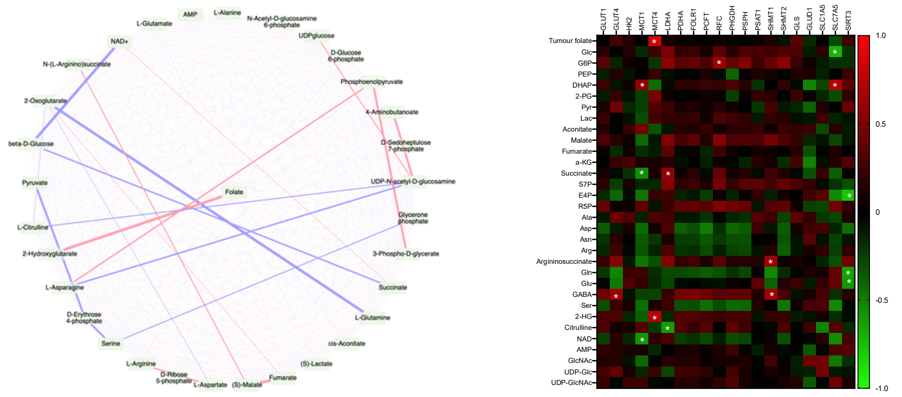

L-Glutamate
AMP
L-Alanine
N-Acetyl-D-glucosamine 6-phosphate
NAD+
UDPglucose
D-Glucose 6-phosphate
N-(L-Arginino)succinate
Phosphoenolpyruvate
2-Oxoglutarate
4-Aminobutanoate
beta-D-Glucose
D-Sedoheptulose 7-phosphate
Pyruvate
UDP-N-acetyl-D-glucosamine
Folate
Glycerone phosphate
L-Citrulline
2-Hydroxyglutarate
3-Phospho-D-glycerate
L-Asparagine
Succinate
D-Erythrose 4-phosphate
L-Glutamine
Serine
cis-Aconitate
L-Arginine
(S)-Lactate
D-Ribose 5-phosphate
Fumarate
L-Aspartate
(S)-Malate
GLUT1
GLUT4
HK2
MCT1
MCT4
LDHA
PDHA
FOLR1
PCFT
RFC
PHGDH
PSPH
PSAT1
SHMT1
SHMT2
GLS
GLUD1
SLC1A5
SLC7A5
SIRT3
Tumour folate
Glc
G6P
PEP
DHAP
2-PG
Pyr
Lac
Aconitate
Malate
Fumarate
a-KG
Succinate
S7P
E4P
R5P
Ala
Asp
Asn
Arg
Argininosuccinate
Gln
Glu
GABA
Ser
2-HG
Citrulline
NAD
AMP
GlcNAc
UDP-Glc
UDP-GlcNAc
1.0
0.5
0
-0.5
-1.0

(B)

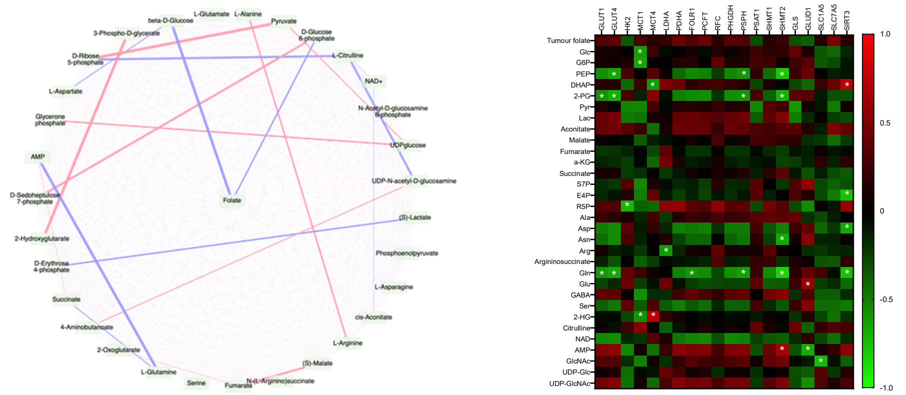

beta-D-Glucose
L-Glutamate
L-Alanine
Pyruvate
3-Phospho-D-glycerate
D-Glucose 6-phosphate
D-Ribose 5-phosphate
L-Citrulline
NAD+
L-Aspartate
N-Acetyl-D-glucosamine 6-phosphate
Glycerone phosphate
UDPglucose
AMP
UDP-N-acetyl-D-glucosamine
D-Sedoheptulose 7-phosphate
Folate
(S)-Lactate
2-Hydroxyglutarate
Phosphoenolpyruvate
D-Erythrose 4-phosphate
L-Asparagine
Succinate
4-Aminobutanoate
cis-Aconitate
2-Oxoglutarate
L-Arginine
(S)-Malate
L-Glutamine
Serine
Fumarate
N-(L-Arginino)succinate
GLUT1
GLUT4
HK2
MCT1
MCT4
LDHA
PDHA
FOLR1
PCFT
RFC
PHGDH
PSPH
PSAT1
SHMT1
SHMT2
GLS
GLUD1
SLC1A5
SLC7A5
SIRT3
Tumour folate
Glc
G6P
PEP
DHAP
2-PG
Pyr
Lac
Aconitate
Malate
Fumarate
a-KG
Succinate
S7P
E4P
R5P
Ala
Asp
Asn
Arg
Argininosuccinate
Gln
Glu
GABA
Ser
2-HG
Citrulline
NAD
AMP
GlcNAc
UDP-Glc
UDP-GlcNAc
1.0
0.5
0
-0.5
-1.0

(C)

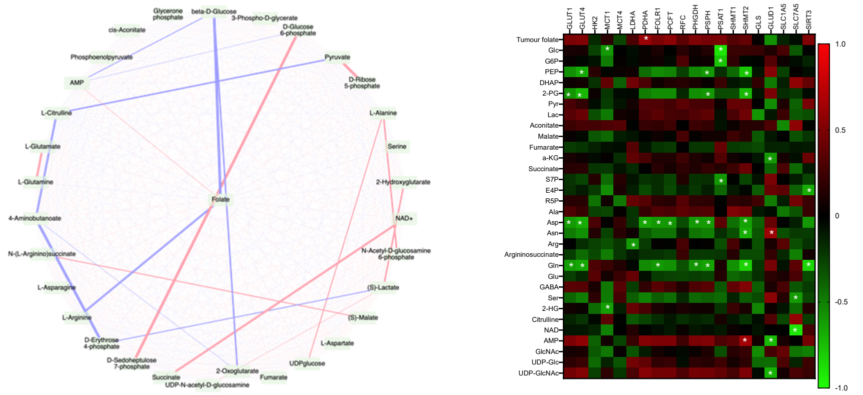

Glycerone phosphate
beta-D-Glucose
3-Phospho-D-glycerate
cis-Aconitate
D-Glucose 6-phosphate
Phosphoenolpyruvate
Pyruvate
D-Ribose 5-phosphate
AMP
L-Citrulline
L-Alanine
L-Glutamate
Serine
L-Glutamine
2-Hydroxyglutarate
Folate
4-Aminobutanoate
NAD+
N-(L-Arginino)succinate
N-Acetyl-D-glucosamine 6-phosphate
L-Asparagine
(S)-Lactate
L-Arginine
(S)-Malate
D-Erythrose 4-phosphate
L-Aspartate
D-Sedoheptulose 7-phosphate
UDPglucose
2-Oxoglutarate
Succinate
Fumarate
UDP-N-acetyl-D-glucosamine
GLUT1
GLUT4
HK2
MCT1
MCT4
LDHA
PDHA
FOLR1
PCFT
RFC
PHGDH
PSPH
PSAT1
SHMT1
SHMT2
GLS
GLUD1
SLC1A5
SLC7A5
SIRT3
Tumour folate
Glc
G6P
PEP
DHAP
2-PG
Pyr
Lac
Aconitate
Malate
Fumarate
a-KG
Succinate
S7P
E4P
R5P
Ala
Asp
Asn
Arg
Argininosuccinate
Gln
Glu
GABA
Ser
2-HG
Citrulline
NAD
AMP
GlcNAc
UDP-Glc
UDP-GlcNAc
1.0
0.5
0
-0.5
-1.0

(D)

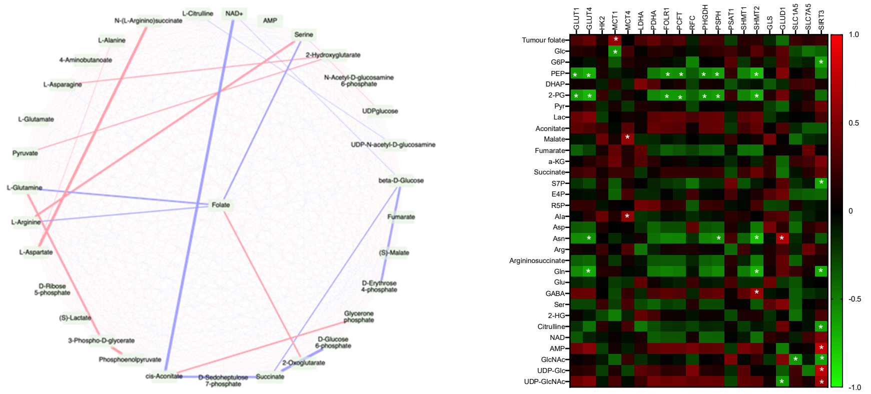

(E)

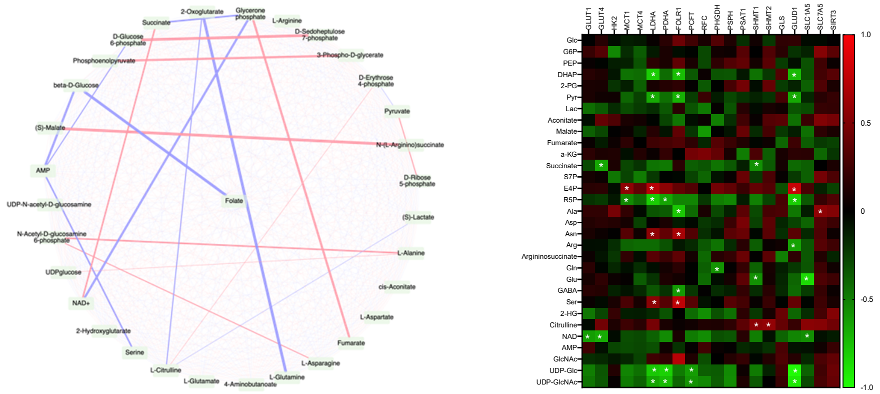

Figure S4. The metabolites and genes interactive network was constructed for tumour pairs as a whole and in the subgroups defined by early stage (A), high tumour folate (B), high plasma folate (C), high folate intake (D), genomic DNA hypermethylation (E).

(A)

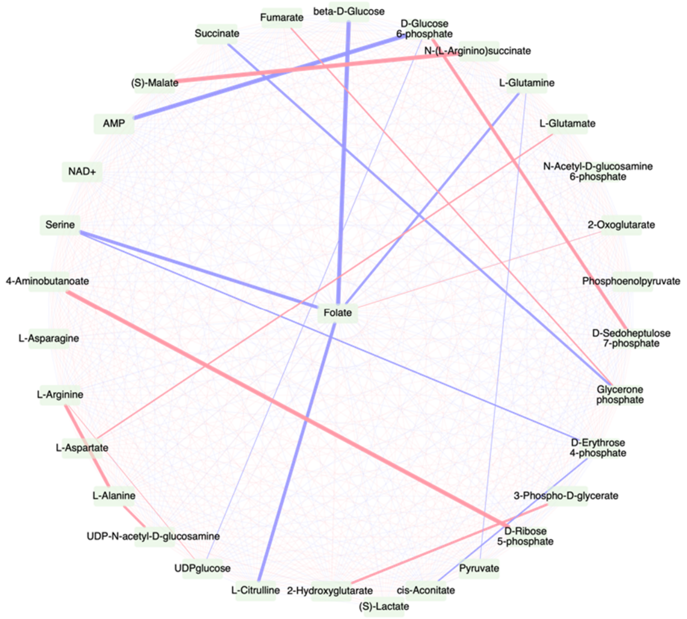
Fumarate
beta-D-Glucose
Succinate
D-Glucose 6-phosphate
N-(L-Arginino)succinate
(S)-Malate
L-Glutamine
AMP
L-Glutamate
NAD+
N-Acetyl-D-glucosamine 6-phosphate
Serine
2-Oxoglutarate
4-Aminobutanoate
Phosphoenolpyruvate
Folate
L-Asparagine
D-Sedoheptulose 7-phosphate
Glycerone phosphate
L-Arginine
D-Erythrose 4-phosphate
L-Aspartate
L-Alanine
3-Phospho-D-glycerate
UDP-N-acetyl-D-glucosamine
D-Ribose 5-phosphate
UDPglucose
Pyruvate
L-Citrulline
2-Hydroxyglutarate
cis-Aconitate
(S)-Lactate

(B)

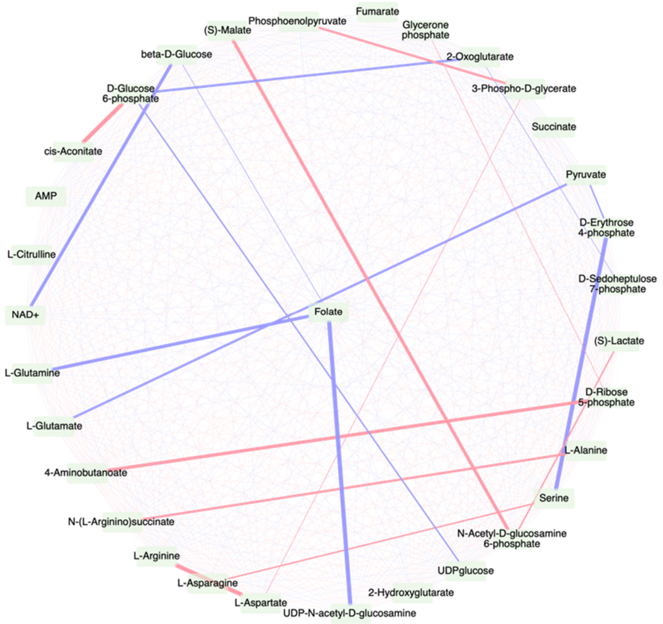
Fumarate
Phosphoenolpyruvate
Glycerone phosphate
(S)-Malate
2-Oxoglutarate
beta-D-Glucose
3-Phospho-D-glycerate
D-Glucose 6-phosphate
Succinate
cis-Aconitate
Pyruvate
AMP
D-Erythrose 4-phosphate
L-Citrulline
D-Sedoheptulose 7-phosphate
NAD+
Folate
(S)-Lactate
L-Glutamine
D-Ribose 5-phosphate
L-Glutamate
L-Alanine
4-Aminobutanoate
Serine
N-(L-Arginino)succinate
N-Acetyl-D-glucosamine 6-phosphate
L-Arginine
UDPglucose
L-Asparagine
2-Hydroxyglutarate
L-Aspartate
UDP-N-acetyl-D-glucosamine

(C)

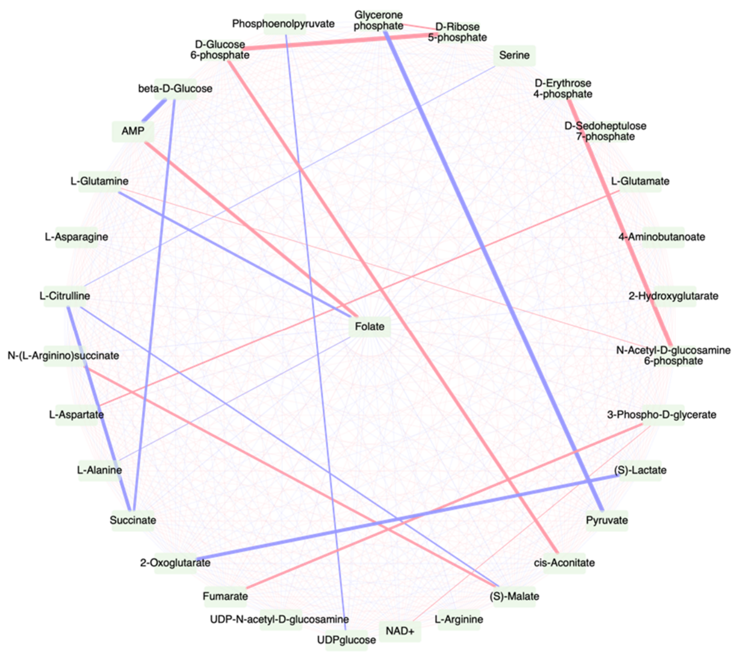

Phosphoenolpyruvate
Glycerone phosphate
D-Ribose 5-phosphate
D-Glucose 6-phosphate
Serine
beta-D-Glucose
D-Erythrose 4-phosphate
AMP
D-Sedoheptulose 7-phosphate
L-Glutamine
L-Glutamate
L-Asparagine
4-Aminobutanoate
L-Citrulline
2-Hydroxyglutarate
Folate
N-(L-Arginino)succinate
N-Acetyl-D-glucosamine 6-phosphate
L-Aspartate
3-Phospho-D-glycerate
L-Alanine
(S)-Lactate
Succinate
Pyruvate
2-Oxoglutarate
cis-Aconitate
Fumarate
(S)-Malate
UDP-N-acetyl-D-glucosamine
NAD+
L-Arginine
UDPglucose

(D)

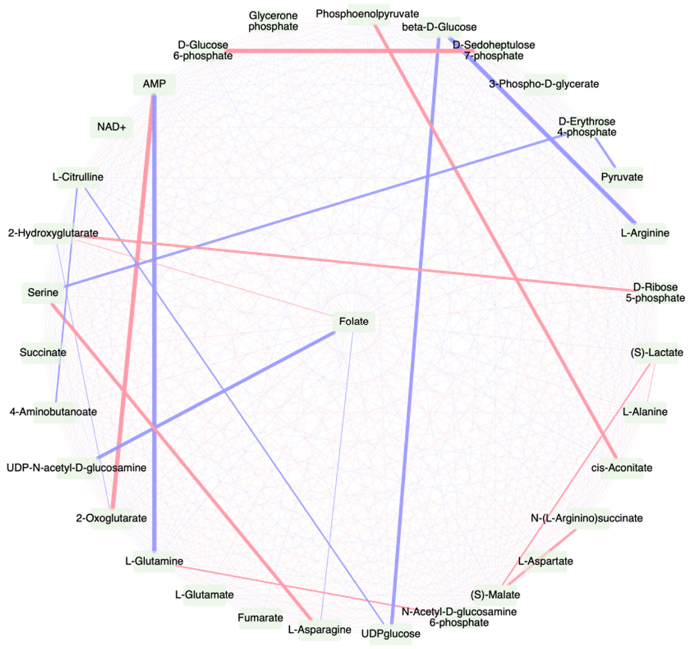

Glycerone phosphate
Phosphoenolpyruvate
beta-D-Glucose
D-Glucose 6-phosphate
D-Sedoheptulose 7-phosphate
AMP
3-Phospho-D-glycerate
NAD+
D-Erythrose 4-phosphate
L-Citrulline
Pyruvate
2-Hydroxyglutarate
L-Arginine
Serine
D-Ribose 5-phosphate
Folate
Succinate
(S)-Lactate
4-Aminobutanoate
L-Alanine
UDP-N-acetyl-D-glucosamine
cis-Aconitate
2-Oxoglutarate
N-(L-Arginino)succinate
L-Glutamine
L-Aspartate
L-Glutamate
(S)-Malate
N-Acetyl-D-glucosamine 6-phosphate
Fumarate
L-Asparagine
UDPglucose

(E)

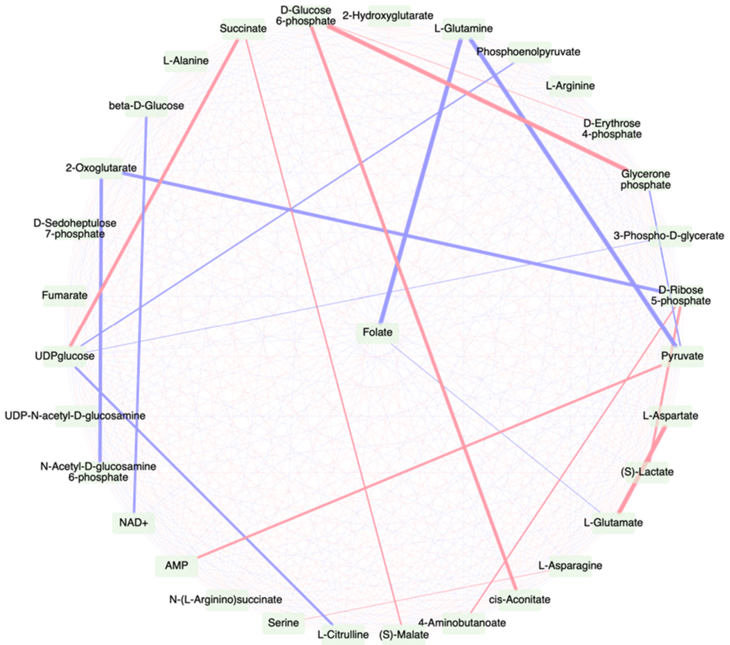

Figure S5. The metabolites and metabolites particle correlation network were constructed for tumour pairs as a whole and in the subgroups defined by advance stage (A), low tumour folate (B), low plasma folate (C), low folate intake (D), genomic DNA hypomethylation (E) For interactive network, edge color and width denote the direction and strength of partial correlations. The red color represents positive correlation; the blue color represents negative correlation. The red color represents positive correlation; the blue color represents negative correlation.

Table S2. The original tissue metabolomics data.

Groups	Glucose	Glucose 6-phosphate	Phosphoenolpyruvate	Dihydroxyacetone phosphate	2-Phosphoglycerate	Pyruvate	Lactate	Aconitate	Malate	Fumarate	a-Ketoglutarate	Succinate	Sedoheptulose 7-phosphate
Non-tumor	54666	601101	101033	83210	79157	70630	1959923	58554	339637	143468	30158	516620	503906
Non-tumor	57893	552242	49564	57212	33060	30197	1017716	23526	134455	91105	27639	921319	403483
Non-tumor	46098	466430	157003	65988	139290	70542	1721645	31779	372329	183086	34086	1560891	486023
Non-tumor	66097	339491	83467	55657	48450	72135	1616780	31152	165889	94942	35672	988606	441933
Non-tumor	60760	511818	205280	111628	180729	71871	1497831	62622	316609	107161	29523	1747269	357886
Non-tumor	55959	376359	40277	48871	29365	46039	1489308	34238	103196	62139	25790	568247	336420
Non-tumor	69403	802026	83225	97064	65047	62885	1857903	73802	242729	97705	33739	853026	614868
Non-tumor	32249	823901	117455	82820	172792	76354	3009605	81821	654630	164696	34676	440477	826226
Non-tumor	94256	716862	111840	60052	102119	48218	1721148	58898	320493	94423	17907	706568	599257
Non-tumor	59367	580455	106312	77132	188651	55199	1874889	45265	399710	108118	30742	1386323	590549
Non-tumor	59912	403578	46124	21418	48463	33198	1661709	42753	324718	126511	65422	544145	372531
Non-tumor	60263	309951	100772	25903	120413	28134	1708028	19463	594537	147528	25503	1872788	450376
Non-tumor	68389	369125	133895	18099	106764	56889	1226827	11940	464732	161016	106908	2318240	410839
Non-tumor	45185	288772	106558	30502	115570	39786	1631427	37379	587422	242245	52122	476664	339079
Non-tumor	67630	634273	119855	22308	137724	41474	1949176	61027	695897	227104	82637	374851	533573
Non-tumor	44975	819066	152802	31714	210102	28258	1836888	63663	701860	166140	16213	483360	837494
Non-tumor	65204	655482	153541	25469	164960	44874	1510326	22355	655074	175440	39000	588035	664455
Non-tumor	68443	583295	89957	11383	92605	43608	1552676	59789	324750	114117	78311	851040	459432
Non-tumor	82277	570250	123818	18491	84043	32505	1798836	11112	374376	207603	51177	386875	468824
Non-tumor	52056	527802	99946	14926	82597	31180	1480211	59063	228066	123153	39949	258806	392712
Non-tumor	71250	468764	31712	14615	44611	26554	1296778	28413	149956	83964	29792	296422	524378
Non-tumor	89315	491825	146123	7615	112193	11422	1892247	18672	661306	190723	32735	821683	451497
Non-tumor	41711	842039	238877	24069	277981	33610	1671044	3047	684343	205211	13997	922759	1172035

Non-tumor	54286	316223	61461	20543	37308	25902	1319375	27860	313599	164453	35221	788187	289711
Non-tumor	40951	419511	95471	28582	159800	33788	1460335	4497	274932	183189	19037	609309	668769
Non-tumor	41771	663106	91625	41472	136953	24064	1392780	10482	425386	176780	15834	636395	932607
Non-tumor	62953	598645	182615	26795	192073	39256	1974416	39057	638642	214762	26897	1012576	664738
Non-tumor	53216	589797	83761	43411	82824	43421	1050250	47424	159222	151955	49811	286709	432682
Tumor	12310	388874	90291	58748	163167	147092	3252005	7592	709652	311088	38593	1366068	724966
Tumor	21863	765536	26301	171413	35006	99416	3660591	31829	667460	396564	64003	445408	639537
Tumor	10947	241502	18391	90087	30276	91071	3592627	21483	498264	248597	46781	655772	308894
Tumor	63727	412359	30297	61099	25977	44783	1956311	32031	75211	50977	27574	828700	251740
Tumor	45964	627927	242503	84965	262687	126489	2644174	14521	578265	165571	31239	663730	587619
Tumor	32012	686540	67943	95382	62100	49435	2384214	52902	183944	114332	20382	204858	523750
Tumor	3767	64931	5472	65767	14488	113009	3809423	21034	1103932	499008	63130	2825815	117520
Tumor	18425	1460445	27347	144343	30773	58479	2995357	64875	621684	204826	27081	248542	1288538
Tumor	17627	1312011	11415	148382	15747	72381	3942651	56986	546365	239121	43255	292946	809848
Tumor	27027	773448	59754	39385	134553	211917	3235787	24678	985260	160570	41174	635483	730181
Tumor	14072	26069	3424	17340	9712	45982	4109956	21983	844930	202100	24544	1865349	87791
Tumor	43035	1751037	57862	26926	162161	46815	2928802	46418	1300395	222150	34524	657449	2286413
Tumor	38616	2523965	21494	62444	51652	33287	3330924	50797	848013	185602	9377	696007	2136411
Tumor	26640	31146	1931	27426	2119	83364	4255415	9442	1194603	146218	50237	3642179	124769
Tumor	45604	494786	143	24441	1994	156109	4536899	37026	1030425	157505	23798	8071606	270813
Tumor	47499	3383137	16623	59319	97568	146077	3872225	16948	2380534	408050	45789	5435947	3101838
Tumor	42582	934179	11600	22037	38361	128716	4196657	36081	2202084	450309	42999	3120808	994220
Tumor	27180	1434997	79246	18447	123392	30751	3092372	41315	693817	148030	7845	437966	1746503
Tumor	39905	649013	122403	16052	429965	171149	3849212	9774	595865	166925	26194	1159633	840309
Tumor	34271	848198	75405	67176	93312	41284	4620386	44293	1167786	390536	23281	578241	610241
Tumor	39380	1941576	65977	30875	103824	36592	3375659	45135	1117260	260270	20451	577066	2434960

Tumor	39123	970158	14967	42053	50044	100013	3933939	9659	856128	245614	40965	2463907	1042881
Tumor	36989	776749	61567	29695	143476	41257	2123921	48439	473152	128926	15848	400919	811676
Tumor	30945	1233822	80530	16525	81835	48815	2464724	53223	435231	121496	4708	355278	1724399
Tumor	30499	1243995	82018	18139	120052	58562	3152607	14581	648088	180429	10883	398557	1688609
Tumor	19805	443194	187	61267	5622	149780	4108323	9090	994454	369199	53945	1356320	580975
Tumor	28996	1605968	39233	25993	60614	86858	3536720	15365	852678	228978	15944	353198	1825250
Tumor	24794	1858234	60944	24928	76480	117825	3445562	40624	630602	233218	35927	483964	3246716

Groups	Erythrose 4-phosphate	Ribose 5-phosphate	Alanine	Aspartate	Asparagine	Arginine	Argininosuccinate	Glutamine	Glutamate	GABA	Serine	2-Hydroxyglutarate	Citrulline	NAD	AMP
Non-tumor	50220	737669	380073	3151238	154531	152590	122041	186543	10999779	8055	1378625	59677	49481	24285	8970
Non-tumor	414300	407228	454355	2878844	137678	126055	74832	210560	10402779	7886	1190296	54614	44957	17452	10978
Non-tumor	36553	653582	533392	3439954	223745	210864	168406	338723	11584548	14609	1635350	55787	29802	34907	8086
Non-tumor	14237	731525	505645	2863334	160237	134305	58558	208592	9989815	9534	1453874	52410	62363	24474	14081
Non-tumor	42398	690245	522788	3210279	233355	190754	142070	262606	11182657	11897	1589517	98095	45231	32454	6992
Non-tumor	9613	480946	464806	2767871	160013	133405	63038	221277	10586061	10072	1202136	27217	46737	9924	3233
Non-tumor	40790	952891	534049	3066075	197149	172454	87293	271554	11052426	9810	1615400	103062	74778	13221	18633
Non-tumor	40872	1238320	439165	3441168	194230	216713	171958	355668	11478061	26867	1210817	120929	47158	38121	16772
Non-tumor	27710	843442	442463	3008965	197269	201990	97962	308055	11286708	11764	1426652	47361	59448	28120	1250
Non-tumor	170274	726372	472771	3053130	162025	186021	140093	200877	10969652	10132	1373786	70519	41511	44977	1606
Non-tumor	8793340	130678	436992	3067685	140247	95642	58586	235978	11399638	13793	1223323	58401	58066	13732	6999
Non-tumor	1655572	135822	529638	3449720	201900	106663	104932	264715	11599984	8885	1519272	77343	49714	45292	6172
Non-tumor	1113587	69439	459239	3001895	165099	100057	64885	228289	11471567	8846	1389523	76290	57763	27453	6122
Non-tumor	310728	120304	478171	3678013	161359	115283	114796	223430	11694243	10102	1193143	130290	43202	27455	5433
Non-tumor	5509998	60329	552640	3365388	226734	103399	129290	196984	11535325	10946	1580405	99797	145728	18984	8916
Non-tumor	2135849	118683	565719	3706969	256479	162458	201832	300713	11861242	14960	1727849	82516	79944	25776	5100
Non-tumor	1194659	82391	514910	3288594	215876	150748	199673	226172	11554305	8258	1569516	85015	47158	21098	6735

Non-tumor	2162289	35126	375136	2758540	221188	101080	81313	229045	10356735	5586	1708888	68929	67035	9439	8556
Non-tumor	6292413	32683	409014	3011325	185376	99659	145418	171975	11023628	8398	1486198	50452	56859	17282	7603
Non-tumor	3229013	30291	423964	3021785	197946	114194	114172	230850	9867196	9483	1509142	57341	98878	11160	5238
Non-tumor	3652066	64519	373535	2763157	120184	103301	65888	213438	10207141	6845	1215149	43276	46414	6966	5227
Non-tumor	3382504	29802	326147	3075651	315214	138609	178849	288907	11206037	4875	2491912	68294	57182	25559	4065
Non-tumor	791913	139510	502574	3521000	227596	140928	222977	263766	11477587	13300	1608273	74420	45563	37474	926
Non-tumor	2522072	68632	485585	3009133	203315	103813	113635	242225	11047785	9200	1570295	63867	40986	21045	4134
Non-tumor	2284806	84895	385860	3232658	110519	124797	110637	224646	11031354	15953	1133800	73950	56019	23604	7578
Non-tumor	3306837	154776	502540	3444063	192020	117371	167727	312795	11408038	13481	1299423	70742	38533	20927	11645
Non-tumor	484810	97072	573326	3493549	243193	169273	194276	329779	11205751	11181	1721930	119609	53647	26257	8744
Non-tumor	1490305	61433	484074	3305588	197526	123634	110954	261535	11165841	13938	1414382	42449	55396	10304	11501
Tumor	36952	314169	548285	3780699	222460	248130	349648	294950	11587380	16570	1424477	203429	26876	128262	45792
Tumor	105572	1311778	538891	3341467	211556	121321	211224	246902	12059193	16116	912966	286606	16314	24165	107231
Tumor	24667	546407	520006	3056025	146415	163025	140673	361183	11161808	12020	853210	82384	27424	7766	50708
Tumor	66868	533528	459638	2383300	118371	92658	33720	196469	10131047	7151	982451	47599	32447	3139	7274
Tumor	32976	582889	549390	3610214	242166	223386	331367	248587	11569108	11077	1605431	155696	37772	35047	23087
Tumor	8661	394495	517329	3098235	168350	189444	151213	260246	11004170	11658	1242898	46691	33731	7783	12884
Tumor	25269	384076	645071	4042297	162665	170685	225749	253057	11479131	15588	1026504	124072	23489	57066	217403
Tumor	97229	1788799	510190	3607045	147785	163934	157367	450362	12107614	20262	882600	118454	49119	14	15831
Tumor	43991	795942	565025	3188054	186032	189079	184276	248380	11604975	18409	886966	120152	23040	2712	56702
Tumor	15503	502675	751973	3562428	245000	273108	531462	202459	11038342	38354	1772672	362528	35041	24692	31717
Tumor	4340163	101204	576231	4460672	166698	197314	215739	269236	11908037	13432	1653092	121084	18398	112849	1197344
Tumor	2743217	314390	730472	3848077	219117	206297	301745	329663	11984986	21403	1695210	128113	32170	8433	5360
Tumor	3458550	778106	423046	3357015	137062	136089	156206	359937	12074359	18813	868676	233936	52089	250	5195
Tumor	1138164	260020	700750	4028453	214709	196723	215534	365463	11999198	18440	1011285	312359	34141	51591	910109
Tumor	882651	623818	726923	3012127	52583	172413	130085	66508	11075571	56207	1015216	171622	20323	13237	1228867

Tumor	2418371	1343977	688789	3840980	224637	269614	492667	261306	11237124	19534	1561017	138441	18127	45821	23290
Tumor	4231561	549799	906700	3583405	375621	178449	480252	215078	12133065	24151	1850885	259976	34701	23198	31326
Tumor	3508039	327537	609984	3749435	276151	238050	309028	304711	11261058	20630	1713331	123852	42611	9389	2094
Tumor	3095575	329810	686597	3691597	167164	196538	165591	257381	11266858	14742	1613875	1192632	23486	47509	4325
Tumor	3926324	496595	689503	3697060	383410	248353	346233	397970	11138275	19049	1666166	225205	48559	20519	25793
Tumor	3863189	581026	619368	3987534	293632	257632	383406	304628	11869196	28144	1616399	358667	39907	25206	6856
Tumor	5510907	418938	455429	3827382	167721	272492	248520	313851	11749439	28585	1765320	136245	16871	23626	20431
Tumor	3937492	107006	558119	3632005	262798	196843	190190	333701	11435052	13444	1525689	60697	34981	17208	921
Tumor	2713058	87318	599008	3723885	269338	197881	227025	322700	11631215	19554	1668800	99113	29198	16528	1064
Tumor	1211485	336405	543812	3660824	189634	131067	220720	340594	11566426	14456	1414047	160805	31010	20295	983
Tumor	300985	403958	750100	3918414	452263	382020	280798	602576	11525028	15105	2330976	168582	38774	45022	352081
Tumor	1284818	386344	614941	3638820	274975	117205	263523	286966	11787043	20871	1101315	109203	27488	17242	22829
Tumor	266202	262814	558366	3913001	326331	162354	241424	348815	12171892	39460	1879108	151353	15668	26935	2364

Groups	N-acetyl-glucosamine	UDP-glucose	UDP-N-acetyl-glucosamine
Non-tumor	2636	43946	4185
Non-tumor	1899	34311	3123
Non-tumor	2381	48653	8088
Non-tumor	2820	47049	2165
Non-tumor	2793	101137	6459
Non-tumor	1829	12204	1779
Non-tumor	4211	37902	5697
Non-tumor	3100	44596	20572
Non-tumor	2113	39603	3239
Non-tumor	4338	26228	4124

Non-tumor	1936	16358	1156
Non-tumor	2866	28441	3000
Non-tumor	2130	38880	3066
Non-tumor	2897	43616	5389
Non-tumor	4047	7139	1789
Non-tumor	3335	17930	3471
Non-tumor	2885	11453	1714
Non-tumor	3488	5294	820
Non-tumor	2906	5725	728
Non-tumor	2654	1828	606
Non-tumor	1884	2082	280
Non-tumor	6164	2825	500
Non-tumor	3269	12024	1313
Non-tumor	2021	5223	633
Non-tumor	2820	5844	73
Non-tumor	2560	3351	675
Non-tumor	4924	16629	2400
Non-tumor	2074	1955	299
Tumor	6006	24516	31958
Tumor	5626	315003	92659
Tumor	3168	221173	97389
Tumor	3051	31010	6856
Tumor	3033	89798	22314
Tumor	5656	10916	9494
Tumor	6649	169726	105590
Tumor	5789	31652	67019

Tumor	6784	61749	91391
Tumor	8229	161579	19483
Tumor	4771	629584	150941
Tumor	7135	32096	17490
Tumor	7093	35853	22053
Tumor	10667	1019359	364615
Tumor	10756	104895	137661
Tumor	7256	9340	35982
Tumor	16915	244541	32436
Tumor	10284	12958	3610
Tumor	7232	71680	11998
Tumor	14120	63477	24458
Tumor	12673	17113	12347
Tumor	11022	153206	25608
Tumor	5119	8915	1373
Tumor	6600	17780	2121
Tumor	7687	12901	3938
Tumor	8470	153069	178125
Tumor	14877	72903	16094
Tumor	5980	14573	10890
